# Imidazolium labelling permits the sensitive mass-spectrometric detection of *N*-glycosides directly from serum†

**DOI:** 10.1039/d1cc02100a

**Published:** 2021-06-17

**Authors:** Yao-Yao Zhang, Mattia Ghirardello, Ting Wang, Ai-Min Lu, Li Liu, Josef Voglmeir, M. Carmen Galan

**Affiliations:** Glycomics and Glycan Bioengineering Research Center (GGBRC), College of Food Science and Technology, Nanjing Agricultural University 1 Weigang 210095 Nanjing China josef.voglmeir@njau.edu.cn www.ggbrc.com; School of Chemistry, University of Bristol Cantock's Close BS8 1TS Bristol UK m.c.galan@bristol.ac.uk www.galanresearch.com; College of Sciences, Nanjing Agricultural University 1 Weigang 210095 Nanjing China

## Abstract

A novel imidazolium derivative (GITag) shows superior ionisation and consequently allows increased mass spectrometric detection capabilities of oligosaccharides and *N*-glycans. Here we demonstrate that human serum samples can be directly labelled by GITag on a MALDI target plate, abrogating prevalently required sample pretreatment or clean-up steps.

## Abstract 2

A novel imidazolium derivative (GITag) shows superior ionisation and therefore allows increased mass spectrometric detection capabilities, herein demonstrated by directly labelling human serum *N*-glycans without sample pretreatment or cleanup steps.

## Graphical abstract

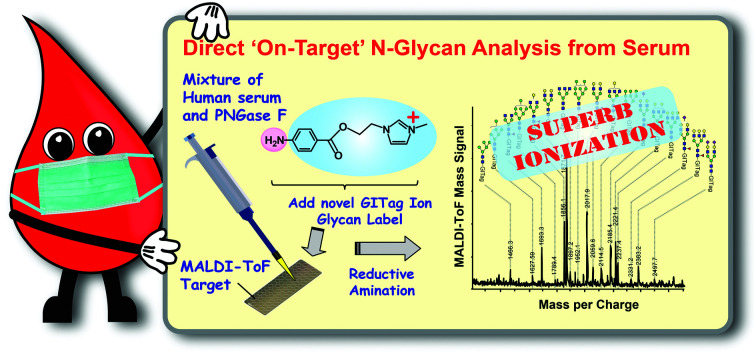

Subtle *N*-glycosylation changes of serum proteins such as immunoglobulins, lipoproteins, or haptoglobin often indicate the transition from a healthy to a pathological state of the body.^1–7^ Alterations of serum *N*-glycosylation were described for various types of cancers, autoimmune diseases, and other diseases,^8–12^ which resulted in the identification of disease-associated *N*-glycosylation signatures.^13–15^ The discovery of glycan biomarkers and the subsequent detection of these altered *N*-glycosylation patterns in clinical implementations require the development of fast and sensitive automatable mass spectrometric analysis methods, with access to a sufficient amount of sample and the analyte limit of detection being one of the main hurdles. In order to address these issues, minimizing or completely avoiding sample enrichment, transfer or centrifugation would be very beneficial in clinical applications (see ESI†). Electrospray ionisation mass spectrometry (ESI-MS) and matrix-assisted laser desorption/ionisation mass spectrometry (MALDI-ToF-MS) provide unique capabilities regarding the sampling throughput and the required analysis times.^16–21^ Currently, the mass spectrometric analysis of total serum *N*-glycans requires (1) an enzymatic or chemical glycan release step, (2) the reductive amination with functionalised primary amine tags, and (3) a sample clean-up step for the isolation of the derivatised *N*-glycan fraction by solid-phase extraction before sample application and analysis.^22–25^ The derivatisation of the reducing end of *N*-glycans with fluorescent or hydrophobic amine tags allows the effective clean-up and/or chromatographic separation and sensitive detection of *N*-glycans in the low femtomol range.^26–28^ 2-Aminobenzamide (2AB) is arguably the most common fluorescent derivatisation tag used in mass spectrometric *N*-glycan analysis and shows similar ionisation efficiencies (within the same magnitude) for ESI-MS and MALDI-ToF as other *N*-glycan derivatisation agents such as 4-aminobenzoic acid 2-diethylaminoethyl ester (procaine), 4-amino-*N*-[(2-diethylamino)ethyl]benzamide (procainamide), 2-amino benzoic acid butyl ester (ABBE) and 2-amino benzoic acid ethyl ester (ABEE).^29^ As an attempt to improve the ionisation efficiency of serum *N*-glycans, the introduction of a permanent charge into the *N*-glycan derivatisation tag was proposed and several elegant attempts to modify oligosaccharides using cationic hydrazides with quaternary ammonia moieties were reported.^30,31^ However, a comparison of the electrospray response of derivatised and native oligosaccharides during mass spectrometric analysis showed only marginal increases in signal intensities in the range of one order of magnitude,^30,31^ which could be attributed to the relatively low derivatisation yields of the oligosaccharides with these types of labels. Nevertheless, the data interpretation was significantly simplified as the ionisation *via* pseudomolecular adduct formation with Group I cations (*e.g.* H^+^, Na^+^, …) is not needed and the [M]^+^ ions could be directly observed.

We previously described the synthesis of various 1-methylimidazolium-tagged glycosides (I-Tags) as excellent probes for mass spectrometric analysis of enzymatic glycosylation reactions even in crude sample matrices, such as milk, since these cationic labels ionised readily and produce a dominant mass spectroscopy signal that can be monitored.^32–34^ Moreover, in a related study we described the synthesis of cationic methylimidazolium *N*-ethylamine (MIEA^+^) and demonstrated that its use in reductive amination reactions of oligosaccharides is feasible.^35^ Although these MIEA^+^-derivatised carbohydrates could be ionised and identified in a proof-of-concept study, attempts to upscale the reductive amination reaction for a more detailed product characterisation failed. Also, a cleanup step after the derivatisation step was required for the removal of the sample matrix and the excess of unreacted MIEA^+^. To ameliorate these shortcomings, we hypothesised that combining an additional aromatic moiety with a methylimidazolium ion would not only provide the stable cationic character required for the efficient mass spectroscopic analysis of the glycan derivatives, but the introduction of an aromatic group facilitates the monitoring of the reaction progress in larger reaction scales due to the chromophoric detection of the reactants *via* UV. This proposed bifunctional imidazolium derivative (GITag, **1**), which carries a permanent positive charge, was successfully synthesised in good yield (74%) by esterification using 4-(Boc-amino)benzoic acid and 1-hydroxylethyl-3-methylImidazolium tetrafluoroborate (Scheme 1).

**Scheme 1:**
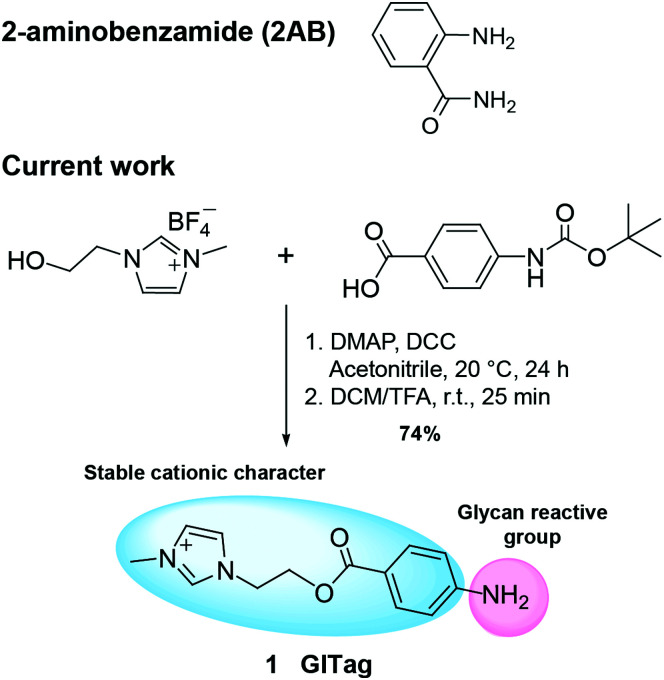
Structures of 2AB and novel GITag and synthetic route for the synthesis of the GITag.

To compare the ionisation efficiency of the GITag **1** with other commonly used tags such as 2AB, the absolute quantification of sample concentrations is essential. Therefore, *N*-acetylglucosamine (GlcNAc, **2**) and lactose (Lac, **3**) were both derivatised *via* reductive amination with GITag and 2AB in a preparative scale and the products obtained in good yields (46–58%) (Scheme 2A, see ESI†). Serial dilutions of these compounds were then subjected to ESI-ToF-LC/MS and MALDI-ToF analysis. The limit of detection (LOD, S/N = 3) and the limit of quantification (LOQ, S/N = 10) of the GITag-labelled compounds **9** and **11** showed up to a 600-fold increase in sensitivity when compared to the 2AB-labeled carbohydrates **8** and **10** for ESI-ToF-LC/MS experiments and by a factor of 2 to 6 when using MALDI-ToF-MS (Table 1). Furthermore, compounds **8–11** showed comparable fluorescence intensities in LC-FLD analysis when using the respective optimised emission and excitation maxima (see ESI†).

**Scheme 2:**
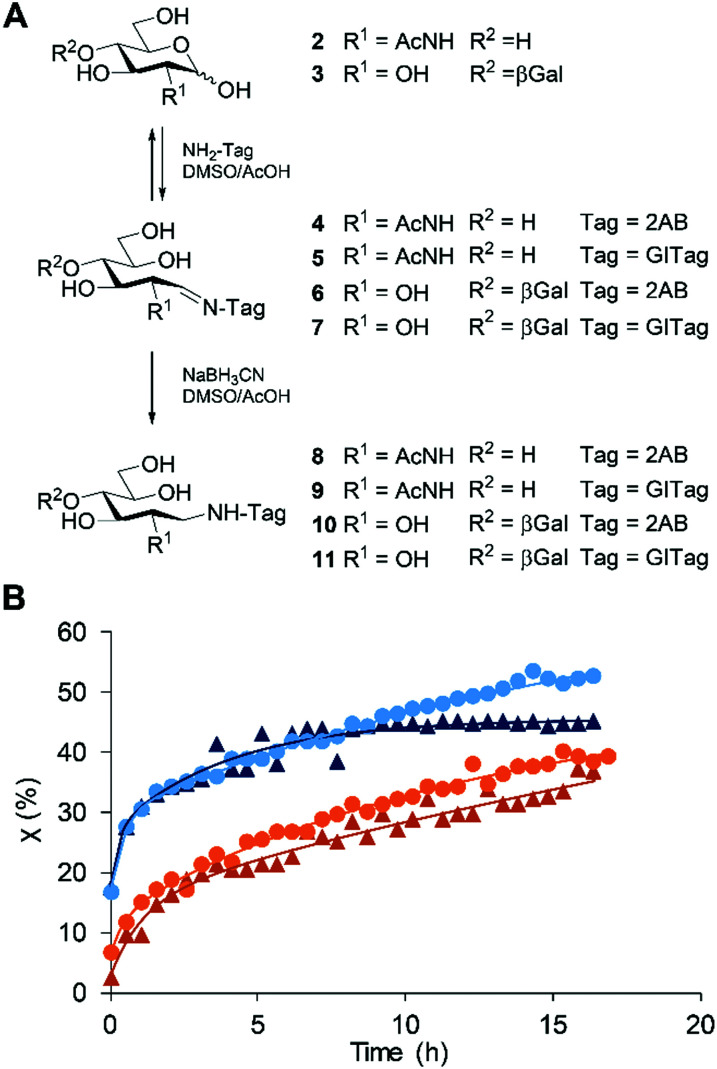
(A) Reductive amination of GlcNAc **2** or Lac **3** with 2AB or GITag and (B), the evolution of the *χ* (%) of compound 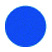 8, 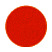 9, 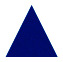 10 and 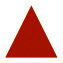 11.

**Table 1:** The limit of detection and quantification of compounds **8–11**

Compound^a^	Fluorescence^a^		ESI-MS		MALDI-ToF-MS	
	LOD (fmol)	LOQ (fmol)	LOD (fmol)	LOQ (fmol)	LOD (fmol)	LOQ (fmol)
2AB-GlcNAc (**8**)	58	189	476	731	779	3576
GITag-GlcNAc (**9**)	229	880	0.7	3.5	104	559
2AB-Lactose (**10)**	3.7	20	1080	2228	686	1402
GITag-Lactose (**11**)	211	602	3.1	9.7	114	685

a Fluorescence intensities were measured for compounds **8** and **10** at Ex/Em wavelengths of 330/420 nm and for compounds **9** and **11** at 304/368 nm.

After establishing the high ionisation efficiency of the GITagged carbohydrates, we also compared the derivatisation efficiency of the reaction of GlcNAc and lactose with GITag **1** and 2AB using NMR spectroscopy (see ESI†). To that end, GlcNAc or Lac were reacted with NaBH_3_CN in the presence of a equimolar amount of 2AB or **1** in a deuterated solvent system (DMSO-*d*_6_/AcOH-*d*_3_ 7 : 3). The conversion rate over time (Scheme 2B) was thus monitored by ^1^H-NMR. Firstly, a faster reaction rate for the monosaccharide GlcNAc was observed when compared to the disaccharide Lac, most probably due to the reduced sterical hindrance of the former. Secondly, for both glycans, neutral 2AB tag results in a slightly faster conversion rate compared to the positively charged GITag. Regardless, in the model system all reactions reach comparable conversions (36 to 52%) within 16 h. We can attribute this rate differences to the cationic nature of **1**, which can interfere with reactions involving other cationic or polar species,^36,37^ and thus is likely to result in slower imine formation (known to be the rate limiting step in reductive amination reactions).^38^ Since the labelling procedure uses an excess of the tag, the small differences in rate did not pose a problem for the efficient functionalization of glycans with **1**, in the same timescale as done for 2AB tagging.

To demonstrate the full potential of GITag **1**, we pursued the challenge of derivatising *N*-glycans from a human serum sample without any pretreatment of the serum or sample clean-up steps following the derivatisation reaction. The human serum sample, PNGase F (the biocatalyst required for the enzymatic *N*-glycan release), sodium phosphate buffer (500 mM, pH 7.5), and the GITag derivatisation solution were deposited sequentially directly on a MALDI-ToF sample carrier without the need for any sample transfer, centrifugation steps (Scheme 3A, see ESI†). All thirty-two major mass signals could be identified as either complex-type (25 species), high mannose-type (5 species), or hybrid-type (2 species) *N*-glycans (Scheme 3B, see ESI†), and showed a comparable glycan distribution to reported human plasma *N*-glycans.^39,40^ It is noteworthy that only neutral *N*-glycans after GITag **1** derivatisation were identified as [M]^+^ ions, whereas single-negative charged monosialylated *N*-glycans had one extra sodium ion attached [M^+−^ + Na^+^]^+^ (*i.e. N*-glycan A2G2S, *m*/*z* = 2331.2), and double-negative charged disialylated *N*-glycans had two extra sodium ions attached [M^+2−^ + 2Na^+^]^+^ (*i.e. N*-glycan A2G2S2, *m*/*z* = 2498.5). The use of the permanently charged GITag **1** allowed for the streamlined determination of sialoglycans without the need of tedious pretreatment of the sample for the neutralization by permetylation or amidation reactions.^41,42^

**Scheme 3:**
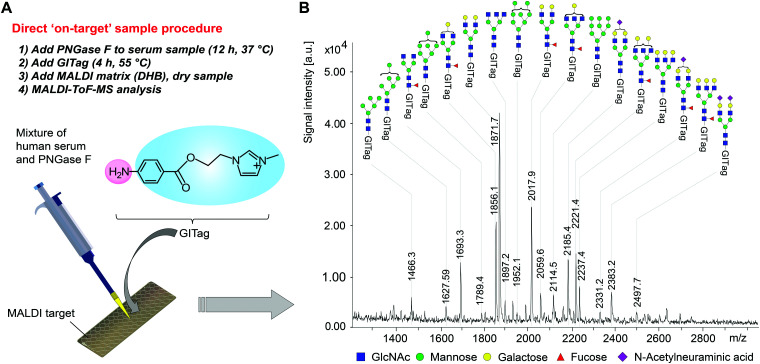
(A) Workflow of human serum *N*-glycan analysis. (B) MALDI-TOF-MS spectra of GITag labelled *N*-glycan from human serum glycoprotein.

To showcase the practical utility of GITag in comparison with commercial 2AB on the detection of human serum *N*-glycans, we undertook *N*-glycan derivatisation with both labels under three different sample preparation set-ups (A) direct on target preparation, (B) in-vial sample preparation with one centrifugation step to remove serum debris after PNGase F treatment, and (C) preparation of samples with a sample clean-up using a solid phase glycan extraction step after PNGase F treatment. In all cases, we clearly showed the better ionisation of GITagged-glycans (see Section 4.1 and Fig. S7 in ESI†). Moreover, we also run a comparison human serum *N*-glycans labelling experiment using sample preparation (C) and the same concentrations of serum and either GITag or 2AB^43^ (see Section 4.2 and Fig. S8 in ESI†). We found that the overall serum *N*-glycan profiles of 2AB-labelled and GI-Tagged serum *N*-glycans were essentially the same, furthermore a direct comparison of the LCMS traces for both labels also showed comparable fluorescence intensities for both 2AB and GITag, with a slightly higher fluorescence at the optimized Ex/Em wavelength of 304/368 nm of the latter. However, as previously demonstrated, when comparing the mass signals for both labelling strategies, significant improvements were observed when using the GITag (∼50 times higher signal intensity when compared to 2AB labelled *N*-glycans). These results further demonstrate the versatility of the new GITag labels.

In conclusion, we have developed a novel glycan MS sequencing label, GITag **1**, bearing both a MS cationic handle and UV/fluorescent-detectable motif, which can be used as an efficient tool for glycan sequencing. The new label is easily accessible in good yields from commercially available starting materials without the need for lengthy purification steps and unlike previously developed cationic labels, carbohydrate derivatisation is efficient and all reactions reach comparable conversions (36 to 52%) within 16 h under standard labelling conditions. More importantly, carbohydrates labelled with the GITag showed superior sensitivity (down to the sub-fmol range) in mass spectrometric analysis when compared to currently used methods. The successful derivatisation of *N*-glycans and analysis of untreated human serum directly on the MALDI sample carrier demonstrates the potential of this derivatisation agent for a broad range of applications in glycan analysis.

This study was funded by the National Natural Science Foundation of China (NSFC) 31471703, 31671854, 31871793 and 31871754 (to JV and LL), and ERC-COG: 648239 (MCG).

## Conflicts of interest

There are no conflicts to declare.

## Supplementary Material

CC-057-D1CC02100A-s001
