# Supplementary material for: Imidazolium labelling permits the sensitive mass-spectrometric detection of N-glycosides directly from serum
Source: Chem Commun (Camb). 2021 Jun 17;57(57):7003–6. doi: 10.1039/d1cc02100a (PMC8280963; doi:10.1039/d1cc02100a)

Electronic Supplementary Material (ESI) for ChemComm.
This journal is © The Royal Society of Chemistry 2021

Supporting Information

Imidazolium derivatisation permits the sensitive mass-spectrometric detection of N-glycosylation directly from serum

Yao-Yao Zhang+, Mattia Ghirardello+, Ting Wang, Ai-Min Lu, Li Liu, Josef Voglmeir*, and M. Carmen Galan*

TABLE OF CONTENTS

1. Material and methods S2

2. Fluorimetric characterization of GITag-Lactose (**11**) S2

3. ESI-ToF-LC/MS and MALDI-ToF analysis and quantifications of 2AB and GITag labelled carbohydrates (**8-11**). S2

4. Application of GITag analysing N-glycans derived from human serum S7

4.1 Comparison of the detection efficiency of human serum N-glycans between carbohydrate tags via MALDI-ToF-MS analysis S10

4.1 Comparison of the detection efficiency of human serum N-glycans between carbohydrate tags via UPLC-FLD-MS analysis S12

5. Comparison of the derivatisation efficiency between carbohydrate tags via ^{1}H NMR analysis S14

6. Preparative scale synthesis and characterization S20

2.1 Synthesis of GITag (**1**) S20

2.2 General reductive amination procedure. S20

2.3 2AB-GlcNAc (**8**) S20

2.4 GITag-GlcNAc (**9**) S20

2.5 2AB-Lac (**10**). S21

2.6 GITag-Lac (**11**) S21

7. ^{1}H-NMR and ^{13}C-APT NMR spectra S23

1. Materials and methods.

1-Hydroxylethyl-3-methylimidazolium tetrafluoroborate was obtained from Energy Chemicals Co. (Nanjing, China); N,N'-dicyclohexylcarbodiimide (DCC) was obtained from Sinopharm Chemical Reagent Co., Ltd. (Shanghai, China); 4-dimethylaminopyridine (DMAP) was obtained from J&K Chemicals Co. (Beijing, China); *N*-methylimidazolium, sodium cyanoborohydride ($NaBH_3CN$), N-acetyl-D-glucosamine (GlcNAc) and Lactose monohydrate were supplied by Aladdin Chemicals Co. (Shanghai, China); Human serum was obtained from Nanjing General Hospital in accordance to the ethical provision number 2017NZKY-023-02. Recombinant PNGase F was expressed and purified as reported previously (Wang et al, *Biosci. Rep.* **2014**, *34*, e00149); Other bulk chemicals were obtained from commercial suppliers without further purification or modification. NMR spectra were registered on a Bruker AV-400 instrument or a Bruker AV-500 instrument using the residual solvent signal as the internal standard at 298 K. NMR data were processed using MestReNova (version 9.0.1). Chromatographic analyses were performed using a Nexera UPLC-FLD system coupled to an LCMS 8040 ESI mass spectrometer (both from Shimadzu Company, Kyoto, Japan). Array NMR experiments were recorded on a Varian 500 MHz spectrometer. High resolution Electrospray ionisation (ESI) mass spectra were recorded on a Micromass LCT mass spectrometer or a VG Quattro mass spectrometer.

2. Fluorimetric characterization of GITag-Lactose (11).

GITag-Lactose (**11**) was dissolved in water (final concentration 10 nM), and the excitation and emission spectra of **11** were recorded on a FluoroMax-4 fluorescence photometer (HORIBA, France). For the emission spectrum, the excitation wavelength was maintained at 350 nm and the emission wavelength was scanned from 325 to 500 nm. For the excitation spectrum, the emission wavelength was kept at 370 nm and the excitation wavelength was scanned from 200 to 350 nm (Figure S1)

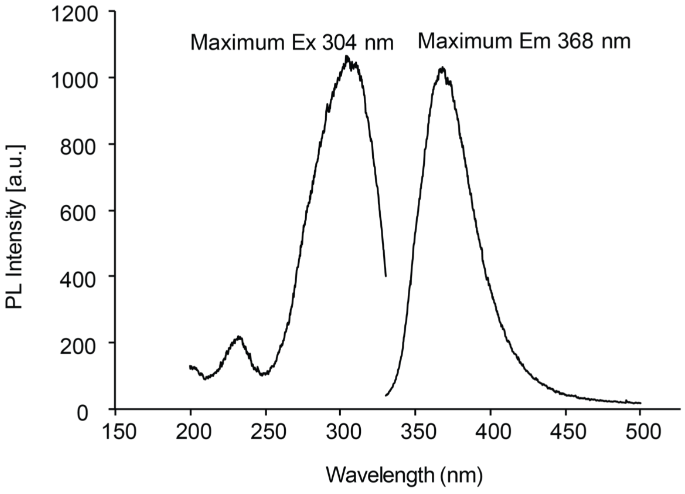

Figure S1. Emission and excitation spectra of GITag-Lactose (**11**).

3. ESI-ToF-LC/MS and MALDI-ToF analysis and quantifications of 2AB and GITag labelled carbohydrates (8-11).

To compare the ionisation efficiency of carbohydrates labelled with the GITag with commonly used derivatisation tags such as 2AB, the absolute quantification of sample concentration based on the gravimetric determination of pure samples is essential. The serial dilutions of 2AB and GITag labelled carbohydrates (range 0.5 nM-500 μM) were then subject to ESI-ToF-LC/MS and MALDI-ToF analysis. Shimadzu LCMS 8040 system (Shimadzu Corporation, Kyoto, Japan) consisting of an LC-30AD pump equipped with a low-pressure gradient mixing unit, a SIL-30AC autosampler, an RF-20Axs fluorescence detector and an ESI mass spectrometric detector. Ten μL of the analytes **8** -**11** were separated on a reversed-phase HPLC column (Phenomenex Hyperclone, 5 μm ODS, 120 Å, 250 × 4.60 mm) at a constant flow rate of 0.8 mL/min with fluorometric detection. Fluorescence

intensities were measured for compounds **8** and **10** at Ex/Em wavelengths of 330/420 nm and for compounds **9** and **11** at 304/368 nm, respectively. Solvent A was 50 mM NH_4COOH (pH 4.5) in water, and solvent B was acetonitrile. A linear gradient of 12−20% solvent B was applied from 0 to 3 min; then, solvent B was increased to 95% over 1 min and held at 95% for 2 min. Solvent B was then decreased to 12% in 1 min, and the column was equilibrated with the initial conditions for 3 min. The compounds **8**, **9**, **10** and **11** appeared at 6.9, 5.7, 5.6 and 4.7 min, respectively. Positive mass signals for **8** ($[M+H]^+$=342.1), **9** ($[M]^+$=451.1), **10** ($[M+Na]^+$=485.0) and **11** ($[M]^+$=572.0) were recorded using the Labsolution LCMS software package (Supporting Figure S2).

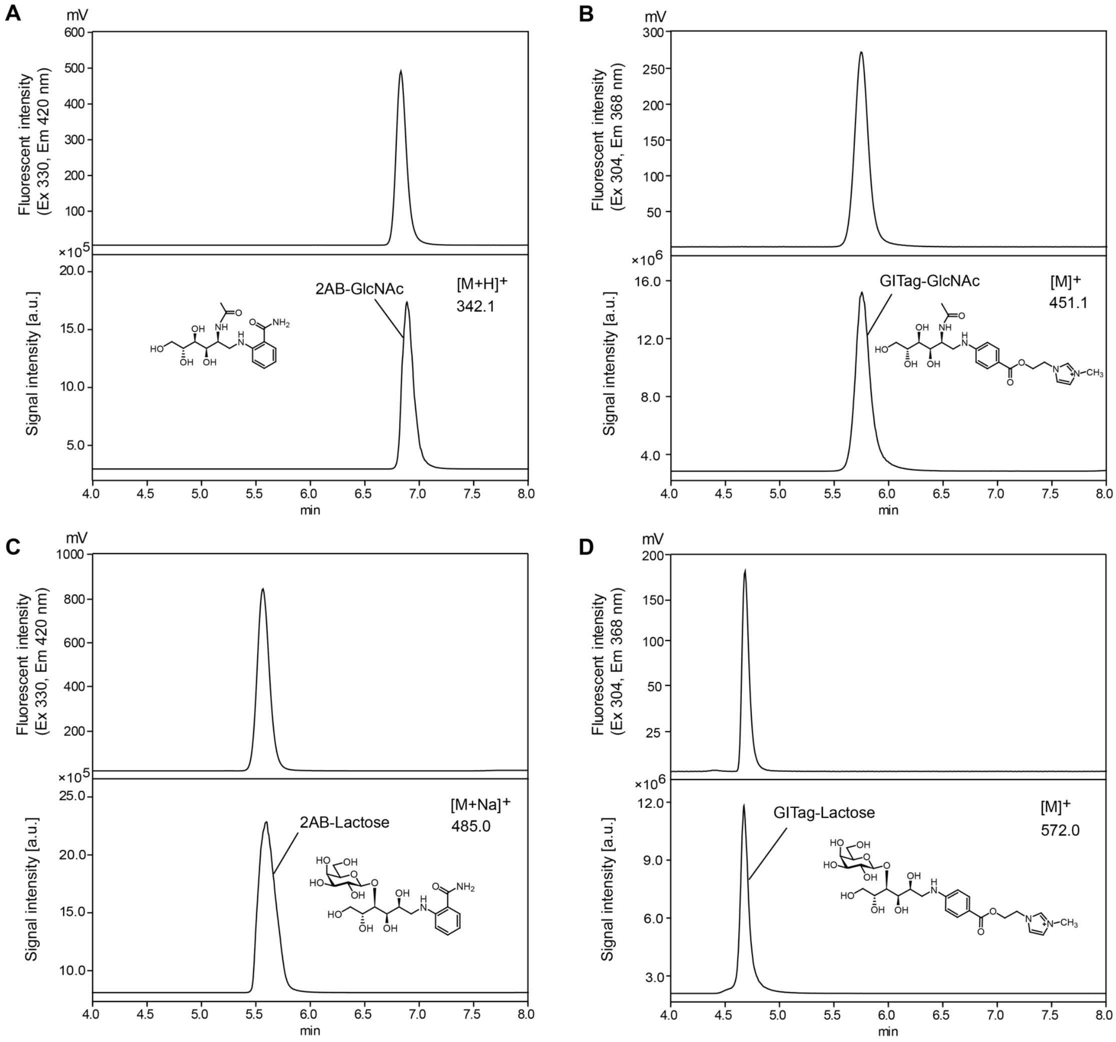

Figure S2. Identification of compounds **8 - 11** by HPLC-FLD-MS analysis: **A** 2AB-GlcNAc (**8**); **B** GlTag-GlcNAc (**9**); **C** ; 2AB-Lactose (**10**); **D** GlTag-Lactose (**11**).

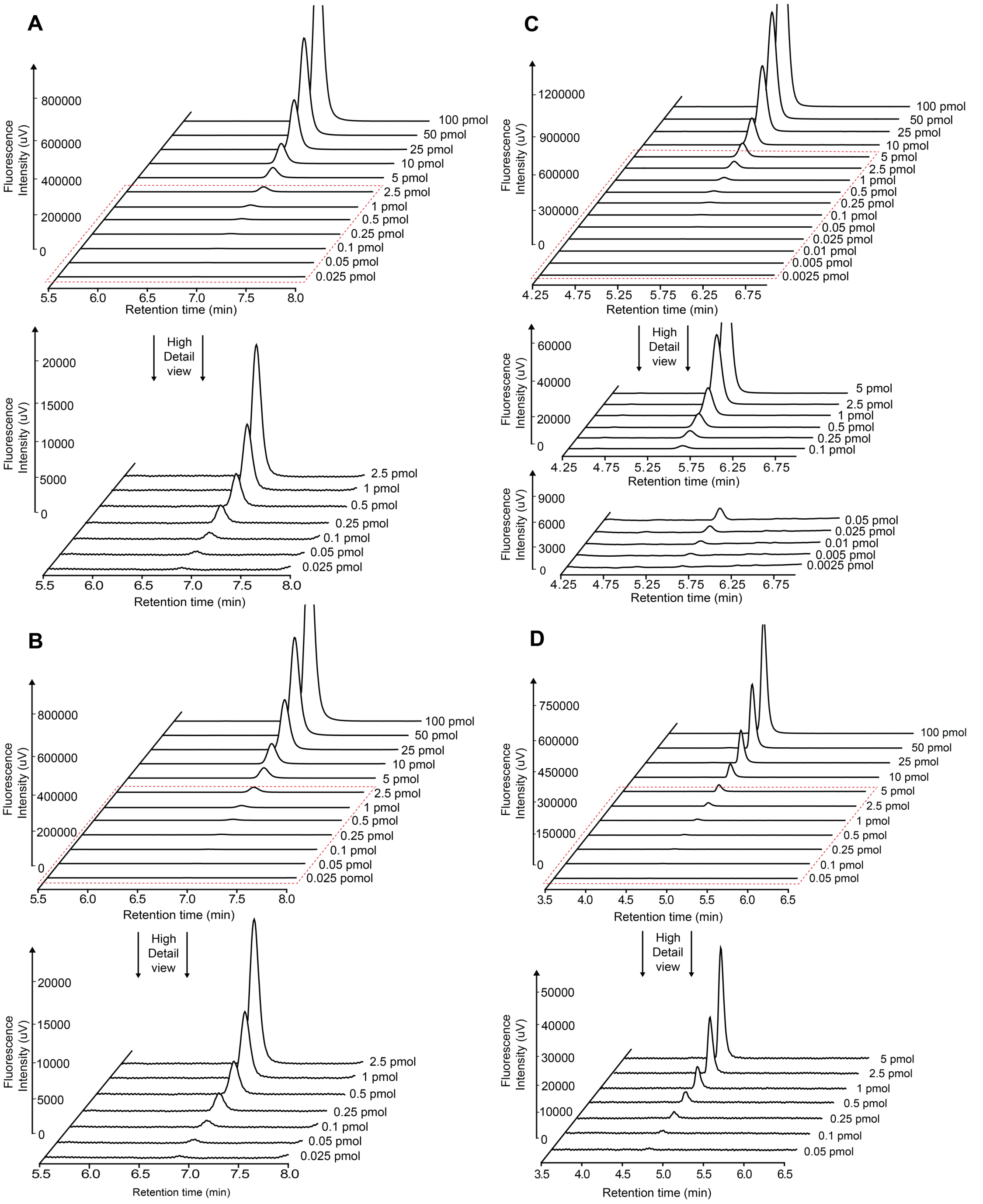

Figure S3. Comparison of fluorescence intensities of compounds **8 – 11** at different concentrations. **A** Fluorescence intensity profile of different concentrations of 2AB-GlcNAc (**8**). **B** Fluorescence intensity profile of different concentrations of GITag-GlcNAc (**9**). **C** Fluorescence intensity profile of different concentrations of 2AB-Lactose (**10**). **D** Fluorescence intensity profile of different concentrations of GITag-Lactose (**11**).

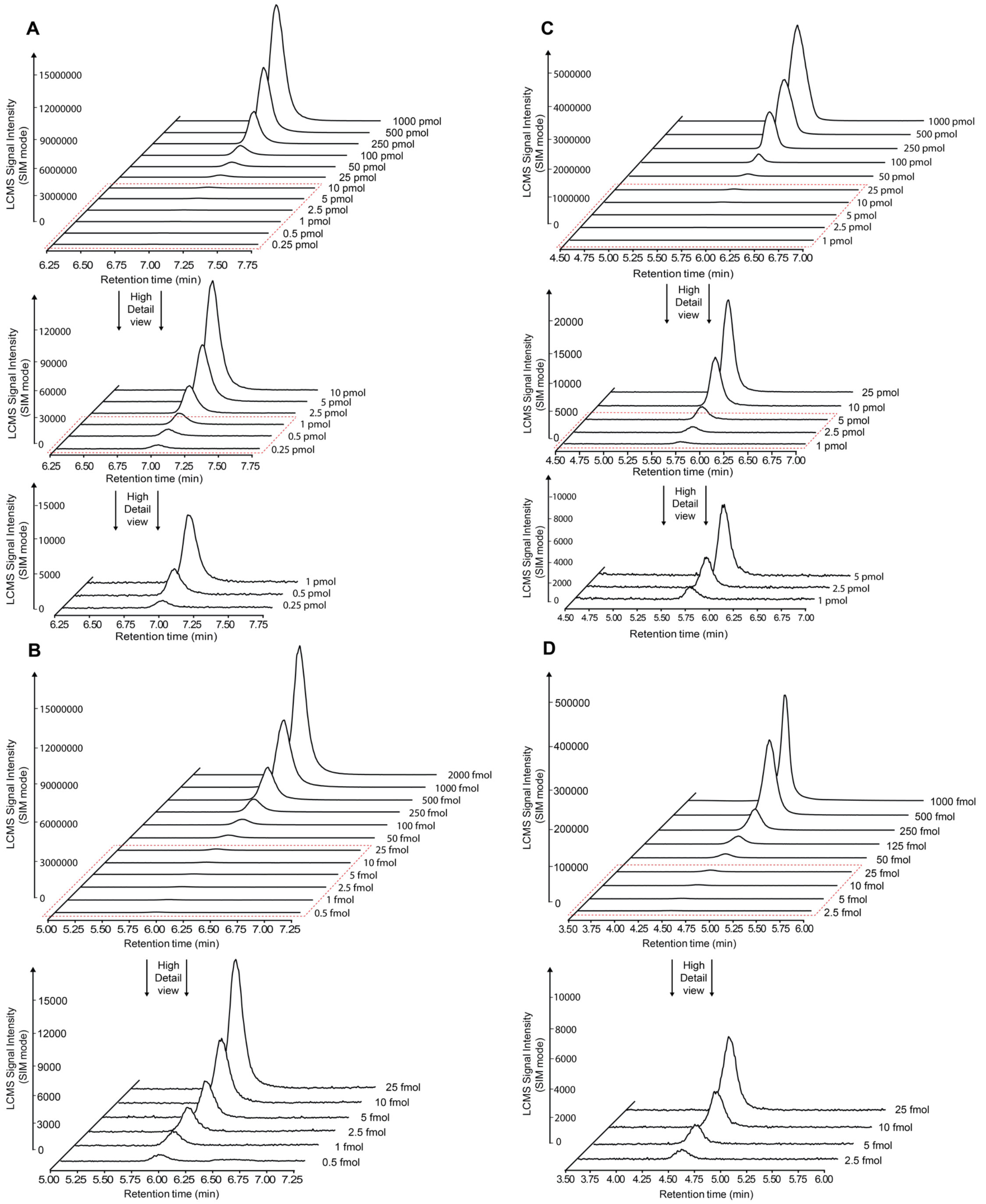

Figure S4. Comparison of extracted ion count (EIC) chromatograms profile of compounds **8 - 11**. **A** EIC chromatogram of different concentrations of 2AB-GlcNAc (**8**). **B** EIC chromatogram of different concentrations of GITag-GlcNAc (**9**). **C** EIC chromatogram of different concentrations of 2AB-Lactose (**10**). **D** EIC chromatogram of different concentrations of GITag-Lactose (**11**).

Serial dilutions of compounds **8 – 11** (1 µL) were spotted on a stain-less-steel MALDI plates and followed by adding 1 µL of 2,5-dihydroxybenzoic acid (DHB) solution (20 mg/ml in 30:70 (v/v) acetonitrile:aqueous TFA (0.1%)). After the samples were dried, they were then analysed on a Bruker Autoflex Speed instrument (equipped with a 1000 Hz Smartbeam-II laser). Mass spectra were analysed by using Bruker Flexanalysis software version 3.3.80 (Supporting Figure S5). The correlation coefficient and linear range are shown in Table S1. The limit of detection (S/N=3) and quantification (S/N=10) of **8 - 11** were determined using the signal intensity areas of the graphs shown in Supporting Figure S3, S4 and S6.

Table S1: Correlation coefficient and linear range of carbohydrates labelled by 2AB and GITag.

Compound	ESI-MS		Fluorescence	
	R^2	Linear range (pmol)	R^2	Linear range (pmol)
2AB-GlcNAc (**8**)	0.9938	0.25-1000	0.9999	0.025-100
GITag-GlcNAc (**9**)	0.9983	0.0005-2	0.9996	0.05-100
2AB-Lactose (**10**)	0.9984	1-1000	0.9999	0.0025-100
GITag-Lactose (**11**)	0.9993	0.0025-1	0.9975	0.05-100

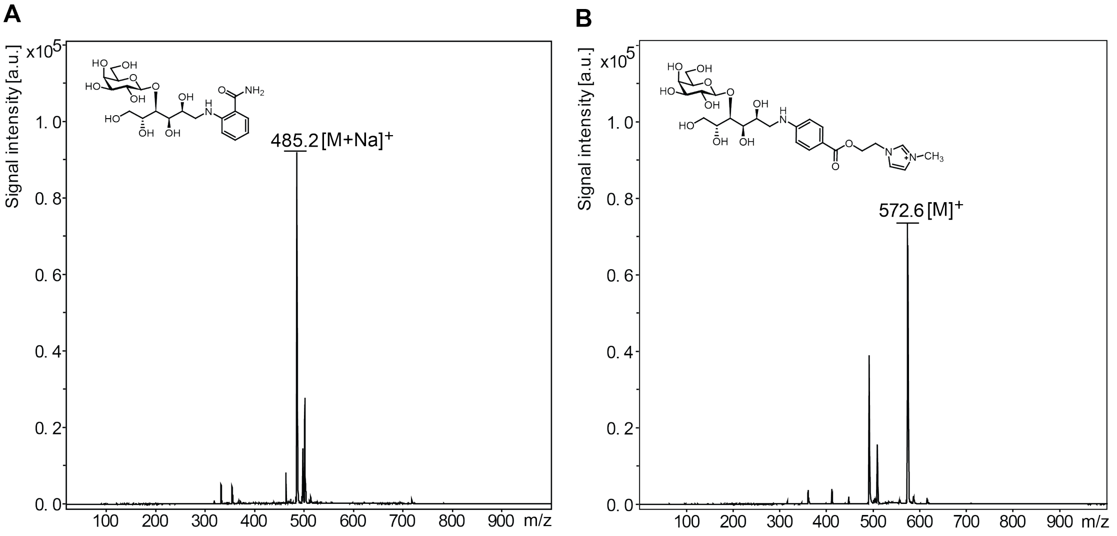

Figure S5. MALDI-ToF-MS spectra of 2AB-Lactose (**10**) and GITag-Lactose (**11**). **A** MALDI-ToF-MS spectra of **10**. **B** MALDI-TOF-MS spectra of **11**.

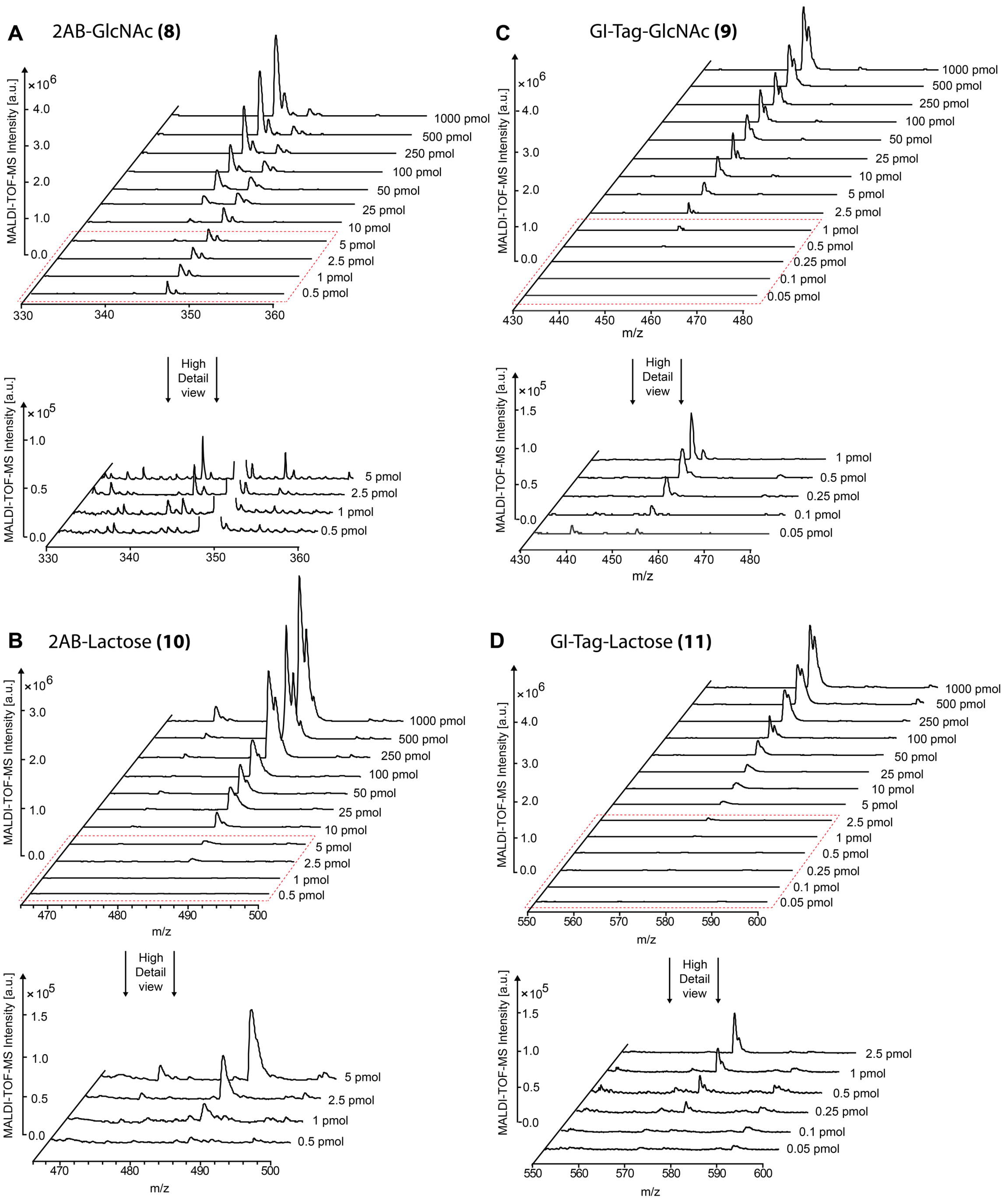

Figure S6. MALDI-TOF-MS spectra of different concentration for compounds **8**-**11** . **A** MALDI-TOF-MS spectra of different concentrations for **8**. **B** MALDI-TOF-MS spectra of different concentration for **10**. **C** MALDI-TOF-MS spectra of different concentration for **9**. **D** MALDI-TOF-MS spectra of different concentration for **11**.

4. Application of GITag analysing N-glycans derived from human serum

Human serum was used without any pretreatment, 50 µL human serum, 60 µL sodium phosphate buffer (500 mM, pH 7.5), recombinant PNGase F (135 µg) and 50 µL GITag derivatisation solution (35 mM GITag and 0.1 M sodium cyanoborohydride in methanol/acetic acid solution (7:3, v/v)) were sequentially added directly on a MALDI-ToF sample carrier (brushed stainless steel) without the need for any sample transfer and centrifugation steps. The MALDI target with the sample mixture was put into a plastic petri dish and incubated at 37 °C for 12 h. Then the MALDI target was transferred to an incubator with the temperature of 55 °C until the sample was dried (4 h). The dried sample was resuspended by 200 µL deionized water, and two µL of the sample transferred on an unused sample spot on the MALDI-Target. After drying and overlaying the samples with 2 µL of DHB matrix (20 mg/ml in 30:70 (v/v) acetonitrile:aqueous TFA (0.1%)), the samples were directly subject to MALDI-ToF mass spectrometric analysis. All thirty-two major mass signals could be identified as either complex- (25 species), high-mannose- (5 species) or hybrid-type (2 species) N-glycans (Supporting Table S2).

Table S2. Summary of GlTag labelled N-glycans from human serum shown in Scheme 3 of the manuscript. ND: not determined.

Name	Structure	Theoretical *m/z* value	Detected m/z value			Glycan type
			$[M]^+$	$[M-H+Na]^+$	$[M-2H+2Na]^+$	
A1	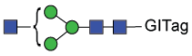	1345.4	1345.1	ND	ND	Complex
M5	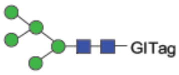	1466.4	1466.8	ND	ND	High mannose
A1G1	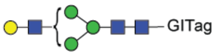	1507.5	1507.1	ND	ND	Complex
A2	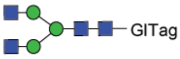	1548.5	1548.7	ND	ND	Complex
M6	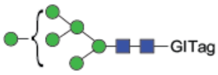	1628.5	1628.1	ND	ND	High mannose
FA1G1	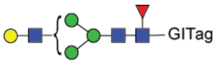	1653.5	1653.6	ND	ND	Complex
FA2	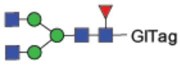	1694.5	1694.5	ND	ND	Complex
A2G1	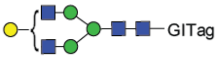	1710.5	1710.9	ND	ND	Complex
M7	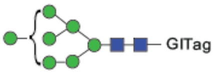	1790.5	1790.3	ND	ND	High mannose
M5A1G1	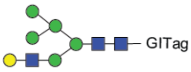	1831.6	1831.2	ND	ND	Hybrid
FA2G1	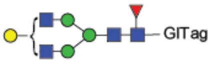	1856.6	1856.3	ND	ND	Complex
A2G2	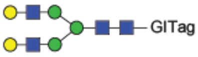	1872.6	1872.1	ND	ND	Complex
FA3	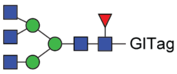	1897.6	1897.4	ND	ND	Complex
A3G1	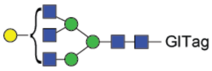	1913.6	1913.9	ND	ND	Complex
M8	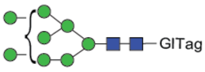	1952.6	1952.1	ND	ND	High mannose
FA2G2	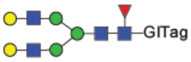	2018.7	2018.2	ND	ND	Complex
FA3G1	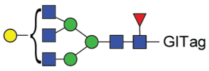	2059.7	2059.6	ND	ND	Complex
A3G2	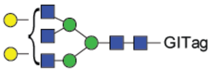	2075.7	2075.8	ND	ND	Complex
M9	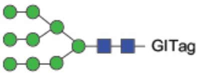	2114.6	2114.5	ND	ND	High mannose

A2G2S	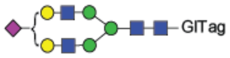	2185.7	ND	2185.5	ND	Complex
FA3G2	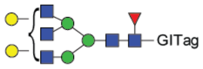	2221.7	2221.3	ND	ND	Complex
A3G3	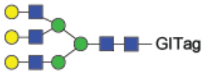	2237.7	2237.9	ND	ND	Complex
FA2G2S	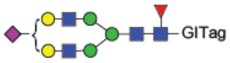	2331.7	ND	2331.2	ND	Complex
FA3G3	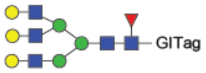	2383.8	2383.2	ND	ND	Complex
A2G2S2	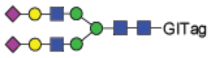	2498.8	ND	ND	2498.5	Complex
FA3G2S	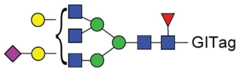	2534.8	ND	2534.4	ND	Complex
A3G3S	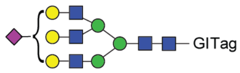	2550.8	ND	2550.7	ND	Complex
A4G4	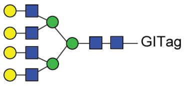	2602.9	2602.7	ND	ND	Complex
FA2G2S2	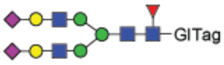	2644.8	ND	ND	2644.3	Complex
FA3G3S	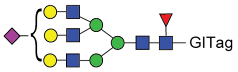	2696.9	ND	2696.0	ND	Complex
A3G3S2	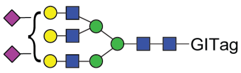	2863.9	ND	ND	2863.1	Complex
FA3G3S2	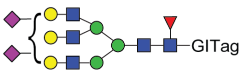	3010.9	ND	ND	3010.7	Complex

4.1 Comparison of the detection efficiency of human serum N-glycans between carbohydrate tags via MALDI-ToF-MS analysis.

In order to compare the detection efficiency of GI-tagged and 2-AB-labelled human serum N-glycans, a set of 3 experiments was performed with the identical derivatisation conditions described for the GI-Tag derivatisation of N-glycans in Section 4 of the SI (35 mM 2-AB labelling solution and a derivatisation conditions of 65 °C/4h were used for the 2AB labelling instead of the 35 mM GITag derivatisation solution and the 55 °C/4h derivatisation conditions used for the GI-Tag.

In a first experiment, the labelling of the serum with 2AB and the GI-Tag was performed without any pretreatment (Figure S7A).

A second derivatisation experiment was not directly performed on a MALDI-ToF sample carrier but in a 1.5 mL sample vial; after the initial enzymatic N-glycan release by PNGase F (37 °C for 12 h), the insoluble part of the sample was removed by centrifugation (14000 g for 5 min). The clear supernatant was then incubated with 50 µL GITag or 2AB derivatisation solution (35 mM GITag or 2AB and 0.1 M sodium cyanoborohydride in methanol/acetic acid solution (7:3, v/v)) and at 55 °C (65 °C for 2AB derivatisation) for 4 h. Two µL of the sample were transferred onto the MALDI-Target, and after drying and overlaying the samples with 2 µL of DHB matrix (20 mg/ml in 30:70 (v/v) acetonitrile:aqueous TFA (0.1%)), the samples were subject to MALDI-ToF mass spectrometric analysis (Figure S7B).

In a third experiment the human serum samples were subject to a solid-phase-extraction (SPE) cleanup-step after the initial

enzymatic N-glycan release; This was achieved by isolating the released N-glycans using ENVI-Carb solid-phase extraction columns (500 mg bed volume, Supelco). These columns were pre-conditioned with 3 ml of deionized water, followed by 3 ml of 80% ACN containing 0.1% TFA (v/v) and finally re-equilibrated with 3 ml of deionized water. Samples containing enzymatically released N-glycans were loaded onto the cartridge and washed with 1.5 ml of water. The N-glycans were eluted using 1.5 ml of 40% acetonitrile containing 0.1% TFA (v/v). Then, samples were dried using centrifugal evaporation. The dried samples were then incubated with either 50 µL GITag or 2AB derivatisation solution (35 mM GITag or 2AB and 0.1 M sodium cyanoborohydride in methanol/acetic acid solution (7:3, v/v)) and at 55 °C (65 °C for 2AB derivatisation) for 4 h. Two µL of the sample were transferred onto the MALDI-Target, and after drying and overlaying the samples with 2 µL of DHB matrix (20 mg/ml in 30:70 (v/v) in aqueous acetonitrile: TFA (0.1%)), the samples were subject to MALDI-ToF mass spectrometric analysis (Figure S7C).

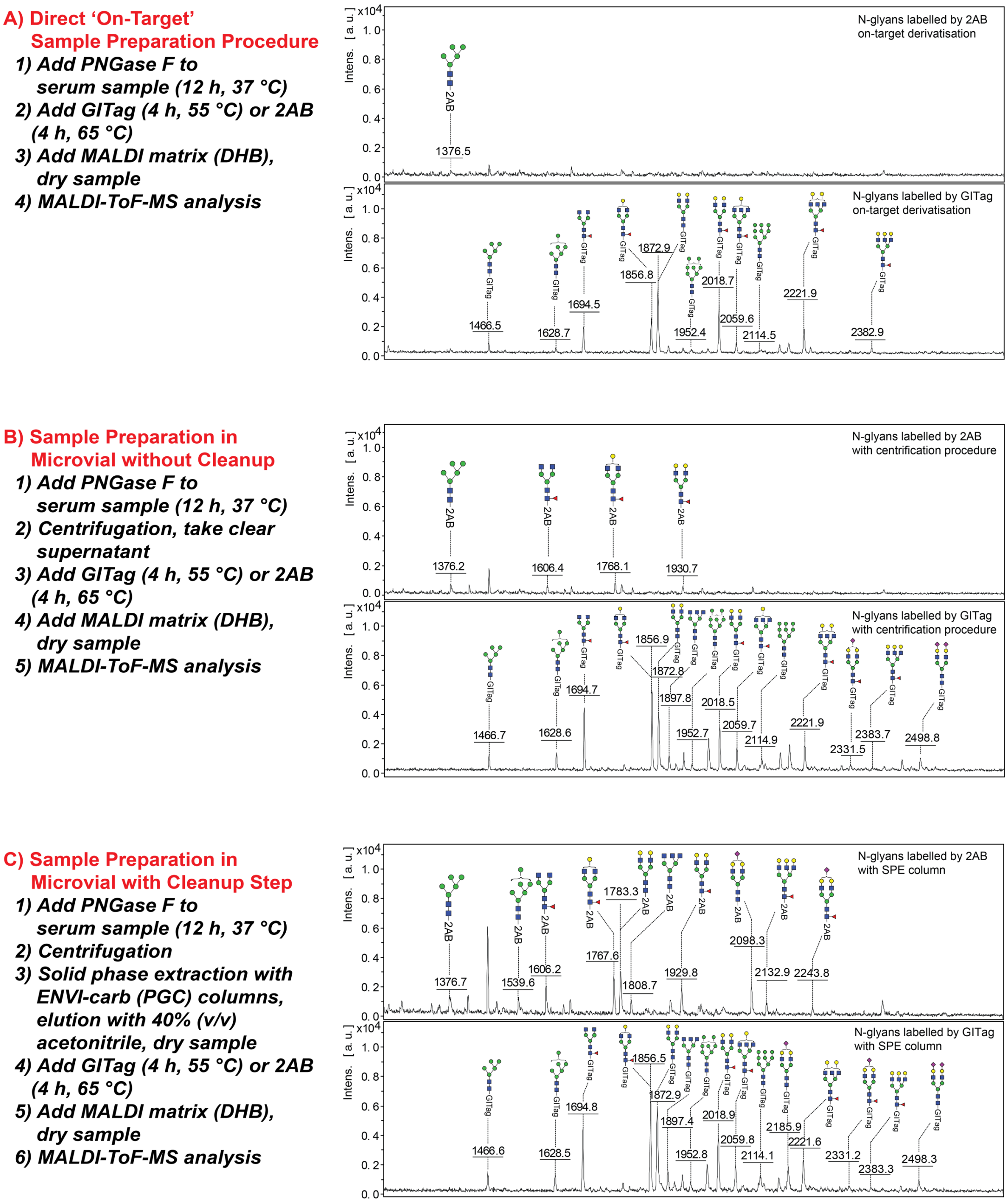

Figure S7. Comparison of the detection efficiency of human serum N-glycans between carbohydrate tags via MALDI-ToF-MS analysis. **A** MALDI-ToF-Spectra from the direct 'On-Target' sample preparation procedure. **B** MALDI-ToF-Spectra from the plasma samples prepared in microvials. **C** MALDI-ToF-Spectra from the plasma samples prepared with a sample cleanup step.

4.2 Comparison of the detection efficiency of human serum N-glycans between carbohydrate tags via UPLC-FLD-MS analysis.

In a third experiment the human serum samples were subject to a solid-phase-extraction (SPE) cleanup-step after the initial enzymatic N-glycan release (Details described in Section 4.1.). The dried serum N-glycans samples were then incubated with either 50 µL GITag or 2AB derivatisation solution (35 mM GITag or 2AB and 0.1 M sodium cyanoborohydride in methanol/acetic acid solution (7:3, v/v)) and at 55 °C (65 °C for 2AB derivatisation) for 4 h. The derivatised N-glycan samples (10 µl) were mixed with 40 µl of acetonitrile. These mixtures were then injected into a UPLC-FLD-MS system (Nexera, Shimadzu Corporation, Kyoto, Japan) and profiled using a hydrophilic interaction liquid chromatography (HILIC) column for the separation of the analytes (Acquity BEH Glycan Column, 2.1×150 mm, 1.7 µm particle size; Waters, Ireland) at a column temperature of 60˚C. The HPLC system consisted of an LC-30AD pump system, an RF-20Axs fluorescence detector set at excitation/emission wavelengths of 330/420 nm for 2AB-labelled N-glycans and 304/368 nm for GI-Tag-labelled N-glycans, respectively. Solvent A was 50 mM aqueous ammonium formate buffer (pH 4.5), and solvent B was acetonitrile. A linear gradient of 95-78% of B was applied from 0 to 6 min, and solvent B was then decreased to 55.9% over 38.5 min, with the flow rate set to 0.5 ml/min. The mass spectrometric analysis was performed using a 8040 ESI-ToF detector using positive SIM mode (Figure S8A-D and Table S3).

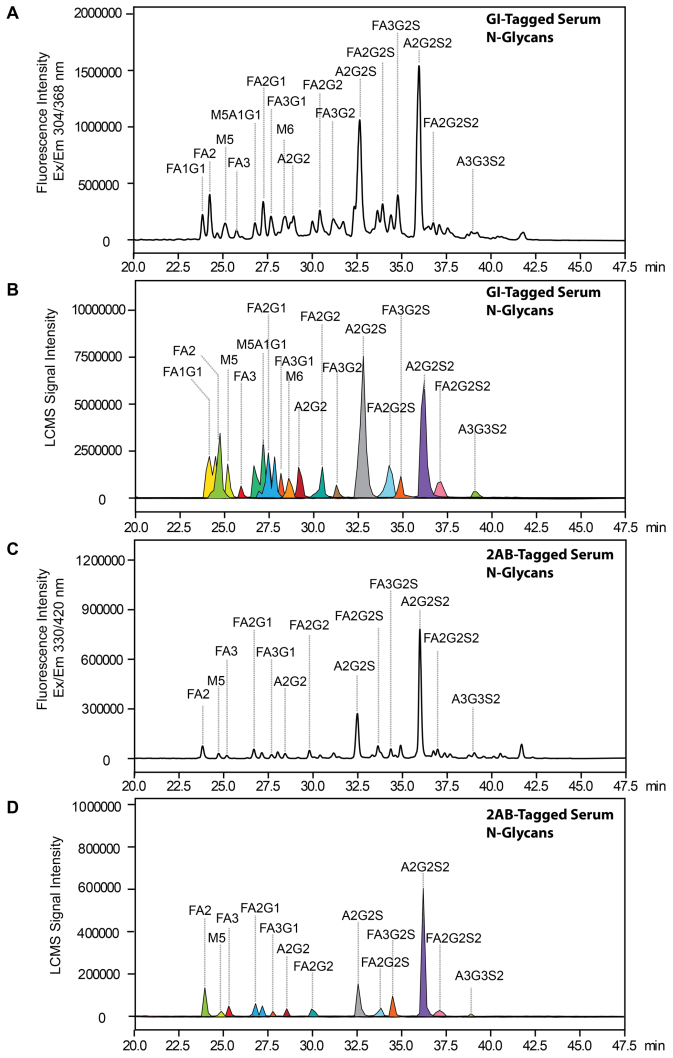

Figure S8. Comparison of the detection efficiency of human serum N-glycans between carbohydrate tags via UPLC-FLD-MS analysis. **A**. Fluorescence detection of UPLC-separated GITagged serum N-glycans at 304/368 nm. **B**. Mass spectrometric detection of UPLC-separated GITagged serum N-glycans. **C**. Fluorescence detection of UPLC-separated 2AB-labelled serum N-glycans at 330/420 nm. **D**. Mass spectrometric detection of UPLC-separated 2AB-labelled serum N-glycans.

Table S3. Summary of the signal intensities obtained from 2AB- and GITag- labelled human serum N-glycans. ND: not detected.

Name	Structure	Signal Intensities				Molecular Weight	
		2AB Fluoresce	GITag Fluorescence	2AB Mass	GITag Mass	2AB	GITag
M5	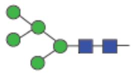	543636	2303243	173824	39852650	1354.5	1466.8
M6	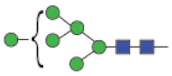	ND	1204093	ND	34260318	1516.5	1628.1
FA1G1	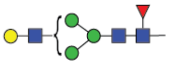	ND	2027874	ND	134948174	1541.5	1653.6
FA2	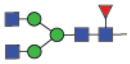	1482757	4094375	2534236	103674591	1582.6	1694.5
M5A1G1	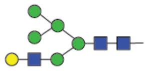	ND	1434591	ND	154181871	1719.6	1831.2
FA2G1	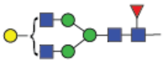	931408	3164591	2231868	99593537	1744.6	1856.6
A2G2	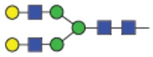	517527	901151	775176	49222519	1760.6	1872.1
FA3	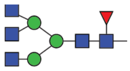	312589	667129	419165	21006831	1785.6	1897.4
FA2G2	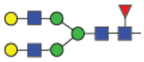	913387	2721425	1054420	46293654	1906.7	2018.2
FA3G1	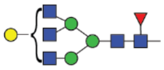	314270	2300898	691273	17662333	1947.7	2059.6
A2G2S	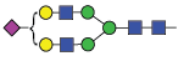	5549192	20783922	3328143	256677177	2051.7	2163.5
FA3G2	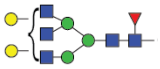	ND	1564136	ND	1165400	1785.6	2221.3
FA2G2S	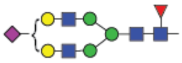	1534255	5160862	914686	78813379	2197.8	2309.2
A2G2S2	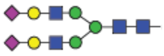	14049428	23585984	10831609	210767058	2342.8	2454.5
FA3G2S	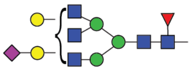	1020601	4027862	1767405	19611749	2400.9	2512.4
FA2G2S2	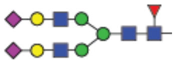	1261257	1023432	1177970	28245697	2488.9	2600.3
A3G3S2	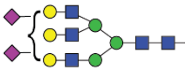	1021088	915807	368327	25933938	2707.9	2819.1

5. Comparison of the derivatisation efficiency between carbohydrate tags via ^{1}H NMR analysis.

In order to compare the derivatisation efficiency of **2AB** and the **GlTag** with carbohydrates, array NMR experiments were performed following the evolution of the reaction over time in a Varian 500 MHz spectrometer, all samples were prepared at the same concentration, using the same batch solvents and conditions. The spectra were acquired at 65 °C with 8 scans at intervals of 30 minutes using the same gain level. The time delay from the additions of reagents into NMR tubes involving sample manipulation and NMR tuning and shimming were minimized as much as possible and the first 10 minutes from each manipulation (addition of tag or addition of NaBH3CN) were not considered.

Sugars, tags and $NaBH_3CN$ were added from stock solutions in DMSO-d_6: GlcNAc (**2**): 28.6 mg/mL; β-Lactose (**3**): 44.2 mg/mL; **2AB**: 39.2 mg/mL; **GlTag**: 56.6 mg/mL and $NaBH_3CN$: 55.7 mg/mL. The same batch of deuterated solvent was used among all the experiments.

The composition of the NMR tube used for each experiment is resumed in table S3.

Table S3. Composition of the NMR tubes.

	**8**	**9**	**10**	**11**
**Sugar**	*GlcNAc*: 10.0 mg (45.2 μmol)	*GlcNAc*: 10.0 mg (45.2 μmol)	β-*Lac*: 15.5 mg (45.2 μmol)	β-*Lac*: 15.5 mg (45.2 μmol)
**Tag**	*2AB* 6.2 mg (45.2 μmol)	*GlTag* 15.1 mg (45.2 μmol)	*2AB* 6.2 mg (45.3 μmol)	*GlTag* 15.1 mg (45.3 μmol)
**$NaBH_3CN$**	8.5 mg (135.6 μmol)	8.5 mg (135.6 μmol)	8.5 mg (135.6 μmol)	8.5 mg (135.6 μmol)

The reductive amination between GlcNAc **2** or Lac **3** with **2AB** or **GlTag** was carried out at 65 °C in a deuterated solvent system composed by DMSO-d_6 and AcOH-d_3 in a 7:3 ratio. The reaction was carried out in a close NMR tube where the chosen sugar at a fixed concentration (Table S3) was allowed to equilibrate with the amine tag to form the corresponding imine before reduction with $NaBH_3CN$.

Conversion % was calculated using DMSO-d_5 residual signal as internal standard. For each experiment the mmol of residual DMSO-d_5 were calculated as a function of the known sugar amount added in each NMR tube (considered as cumulative integration of the α and β H-1 signals) as:

$$mmol_{DMSO} = \frac{mmol_{Sug} \cdot I_{DMSO}}{I_\alpha + I_\beta}$$

Where $mmol_{DMSO}$ are the mmoles of residual DMSO-d_5, $mmol_{Sug}$ are the mmoles of the corresponding sugar present in the NMR tube, I_{DMSO} is the integral of the residual DMSO-d_5 signal, I_α is the integral of the H-1 α anomeric signal and I_β is the integral of the H-1 β anomeric signal. The integration ranges are listed in table S4.

Table S4. 500 MHz ppm range for H-1 signals for α and β GlcNAc and Lac in DMSO-d_6/AcOH-d_3 7:3 at 65 °C

H-1, compound	ppm
**α-D-GlcNAc**	*5.00-4.92*
**β-D-GlcNAc**	*4.49-4.40*
**α-D-Lac**	4.94-4.89
**β-D-Lac**	4.37-4.31

$mmol_{DMSO}$ was normalized to the initial volume of deuterated DMSO used:

$$mmol_{DMSO/mL} = \frac{mmol_{DMSO}}{mL}$$

In each NMR spectrum the conversion (χ %) to the product is expressed as function of I_{DMSO} used as internal standard taking into account the addition volumes of the tag and $NaBH_3CN$ dissolved in DMSO-d_6 as:

$$x\ (\%) = \frac{mmol_P \cdot 100}{mmol_{Sug}} = \frac{I_P \cdot \frac{mmol_{DMSO}}{I_{DMSO}} \cdot 100}{mmol_{Sug}} = \frac{I_P \cdot \frac{mmol_{DMSO/mL} \cdot mL_{DMSO}}{I_{DMSO}} \cdot 100}{mmol_{Sug}}$$

Where I_P is the integral of the H-2 signal of the product (listed in table S5) and mL_{DMSO} is the total volume of deuterated DMSO in the sample after tag and $NaBH_3CN$ addition.

Table S5. 500 MHz ppm range for H-2 signals for **8-11** in DMSO-d_6/AcOH-d_3 7:3 at 65 °C

H-2, compound	ppm
**8**	4.14-4.02
**9**	4.11-3.99
**10**	3.89-3.83
**11**	3.98-3.90

350 μL of the DMSO-d_6 solution of sugar were mixed with 150 μL of AcOH-d_3, the 1 H spectrum was recorded, the T was raised to 65 °C and the tube was allowed to equilibrate for 500 seconds spinning at 20 Hz before acquisition of a new 1H spectrum. The tube was quickly removed, 1 equivalent of tag was added, the tube was placed again in the spectrometer at a constant T of 65°C and an array of 1H spectra was recorded at intervals of 30 minutes. After 6 hours the tube was quickly removed, 3 equivalents of $NaBH_3CN$ were added the tube was placed again in the spectrometer at a constant T of 65°C and an array of 1H spectra was recorded at intervals of 30 minutes. Integral values (arbitrary unit) are listed below in table S6-9.

Table S6. 500 MHz ^{1}H integral values (arbitrary unit) for the study of the reductive amination between **2** and **2AB**.

	time (h)	***8** H-2 integral*	*DMSO-d_5 integral*	
		4.144-4.020 ppm	2.505-2.461 ppm	χ (%)
1	0,001	255,864	1124,320	16,759
2	0,512	426,317	1146,250	27,389
3	1,024	479,081	1164,380	30,299
4	1,536	525,567	1166,530	33,178
5	2,047	539,756	1169,870	33,977
6	2,559	561,422	1176,520	35,141
7	3,071	582,703	1180,550	36,348
8	3,583	566,912	1171,950	35,623
9	4,094	614,802	1173,380	38,585
10	4,606	612,825	1171,700	38,516
11	5,118	615,093	1171,710	38,658
12	5,630	636,768	1172,520	39,993
13	6,141	660,641	1171,900	41,514
14	6,653	659,011	1169,200	41,507
15	7,165	660,997	1168,700	41,650
16	7,676	675,572	1168,000	42,594
17	8,188	706,894	1168,310	44,557
18	8,700	699,153	1167,640	44,094
19	9,212	730,187	1169,970	45,960
20	9,723	734,315	1168,490	46,278
21	10,235	746,017	1169,310	46,983
22	10,747	755,827	1169,130	47,608
23	11,259	759,581	1168,640	47,865
24	11,770	772,891	1168,280	48,718
25	12,282	779,858	1168,140	49,163
26	12,794	786,596	1169,380	49,536
27	13,305	799,777	1168,780	50,391
28	13,817	819,878	1167,030	51,735
29	14,329	847,171	1170,550	53,297
30	14,841	824,707	1169,170	51,945
31	15,352	814,745	1168,660	51,340
32	15,864	825,481	1170,440	51,937
33	16,376	832,217	1171,390	52,319

Table S7. 500 MHz ^{1}H integral values (arbitrary unit) for the study of the reductive amination between **2** and **GlTag.**

	time (h)	***9** H-2 integral*	*DMSO-d$_5$ integral*	
		4.105-3.993 ppm	2.508-2.457 ppm	χ (%)
1	0,001	94,858	1124,120	6,453
2	0,512	169,059	1111,620	11,631
3	1,024	215,962	1115,290	14,809
4	1,536	247,117	1106,300	17,083
5	2,047	269,737	1105,230	18,665
6	2,559	243,098	1107,780	16,783
7	3,071	308,579	1109,710	21,266
8	3,583	330,587	1109,960	22,778
9	4,094	313,312	1112,640	21,536
10	4,606	365,060	1119,070	24,948
11	5,118	370,422	1109,280	25,538
12	5,630	383,615	1104,550	26,561
13	6,141	385,242	1099,560	26,795
14	6,653	383,151	1098,690	26,670
15	7,165	412,791	1096,240	28,798
16	7,676	422,701	1100,500	29,375
17	8,188	446,975	1101,150	31,043
18	8,700	428,569	1092,780	29,993
19	9,212	444,184	1092,390	31,097
20	9,723	456,607	1090,230	32,030
21	10,235	459,995	1089,030	32,303
22	10,747	485,276	1092,150	33,981
23	11,259	484,182	1099,640	33,674
24	11,770	490,752	1097,190	34,207
25	12,282	539,544	1085,490	38,013
26	12,794	488,264	1078,630	34,619
27	13,305	509,914	1080,060	36,106
28	13,817	529,683	1078,860	37,548
29	14,329	528,611	1079,090	37,464
30	14,841	530,790	1071,520	37,884
31	15,352	557,647	1068,160	39,926
32	15,864	543,756	1063,660	39,096
33	16,376	527,777	1057,480	38,169
34	16,887	538,309	1051,630	39,147

Table S8. 500 MHz ^{1}H integral values (arbitrary unit) for the study of the reductive amination between **3** and **2AB**.

	time (h)	***10** H-2 integral*	*DMSO-d$_5$ integral*	
		3.886-3.828 ppm	2.499-2.464 ppm	χ (%)
1	0,001	253,058	1094,470	17,419
2	0,512	377,065	1038,280	27,359
3	1,024	428,842	1043,750	30,953
4	1,536	457,345	1047,420	32,894
5	2,047	477,000	1048,390	34,276
6	2,559	484,134	1051,050	34,701
7	3,071	499,093	1057,240	35,564
8	3,583	578,432	1061,190	41,064
9	4,094	517,021	1053,980	36,955
10	4,606	521,796	1056,900	37,193
11	5,118	602,097	1053,570	43,053
12	5,630	530,631	1049,660	38,084
13	6,141	600,697	1054,840	42,901
14	6,653	611,243	1056,920	43,568
15	7,165	614,150	1058,660	43,704
16	7,676	541,413	1066,590	38,241
17	8,188	617,892	1060,000	43,914
18	8,700	616,375	1054,080	44,052
19	9,212	622,825	1057,130	44,385
20	9,723	624,270	1054,630	44,593
21	10,235	627,757	1060,670	44,587
22	10,747	627,069	1069,340	44,177
23	11,259	627,428	1052,640	44,904
24	11,770	629,583	1055,760	44,925
25	12,282	628,226	1058,350	44,718
26	12,794	628,934	1054,570	44,929
27	13,305	629,351	1053,560	45,002
28	13,817	628,360	1060,130	44,653
29	14,329	627,241	1049,310	45,033
30	14,841	626,382	1064,850	44,315
31	15,352	626,587	1060,140	44,526
32	15,864	627,646	1059,350	44,635
33	16,376	633,216	1055,900	45,178

Table S9. 500 MHz ^{1}H integral values (arbitrary unit) for the study of the reductive amination between **3** and **GITag**.

	time (h)	***11** H-2 integral*	*DMSO-d_5 integral*	
		3.980-3.899 ppm	2.499-2.464 ppm	χ (%)
1	0,001	30,056	1084,850	2,190
2	0,512	127,631	1062,110	9,498
3	1,024	126,841	1062,640	9,435
4	1,536	198,469	1073,570	14,612
5	2,047	217,445	1077,490	15,951
6	2,559	253,825	1083,120	18,523
7	3,071	267,228	1088,170	19,411
8	3,583	294,360	1097,140	21,207
9	4,094	280,826	1096,440	20,244
10	4,606	281,800	1101,910	20,214
11	5,118	293,959	1101,290	21,098
12	5,630	293,255	1099,570	21,080
13	6,141	312,280	1097,800	22,484
14	6,653	375,482	1112,200	26,685
15	7,165	359,130	1094,880	25,926
16	7,676	342,047	1093,070	24,734
17	8,188	387,041	1088,870	28,095
18	8,700	355,451	1090,440	25,765
19	9,212	408,239	1088,580	29,642
20	9,723	371,739	1086,800	27,036
21	10,235	396,307	1091,340	28,703
22	10,747	443,847	1089,180	32,210
23	11,259	394,220	1080,130	28,848
24	11,770	404,972	1090,360	29,357
25	12,282	404,816	1077,690	29,691
26	12,794	455,413	1070,680	33,620
27	13,305	425,755	1078,020	31,217
28	13,817	420,412	1071,410	31,015
29	14,329	435,435	1072,220	32,099
30	14,841	443,695	1079,660	32,483
31	15,352	448,850	1072,230	33,088
32	15,864	498,954	1069,850	36,863
33	16,376	504,887	1084,430	36,800

6. Preparative scale synthesis and characterization.

6.1 Synthesis of GlTag (1)

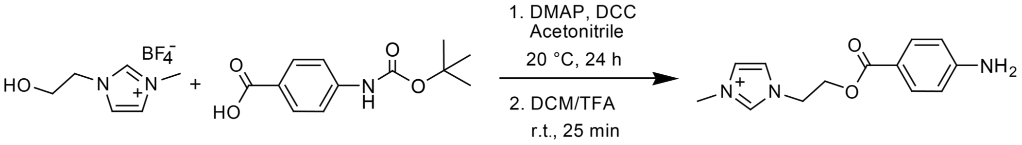

DCC (619.0 mg, 3.0 mmol) was added to a reaction mixture of DMAP (36.7 mg, 0.3 mmol), and 1-hydroxylethyl-3-methyl imidazolium tetrafluoroborate (667.6 mg, 3.12 mmol) in acetonitrile (25 mL) and stirred at room temperature for 10 minutes. 4-(boc-amino)benzoic acid (740,3 mg, 3.12 mmol) was added and the solution was stirred at room temperature for 24 h. The white precipitate of dicyclohexylurea was removed by filtration and the clear filtrate solution was concentrated under reduced pressure by rotary evaporation. The residue was triturated with diethyl ether several times until TLC analysis shows complete removal of the starting materials. The residue was dissolved in 5 ml of trifluoroacetic acid/dichloromethane 1:1 (v/v) solution and stirred at room temperature for 20 min. The reaction mixture was concentrated under reduced pressure by rotary evaporation and the liquid residue was co-evaporated three times with 5 ml methanol to remove trifluoroacetic acid residues furnishing **1** (769.0 mg, 74 %) as a yellow oil.

^{1}H NMR (500 MHz, D_2O, 25 °C) δ 8.83 (d, 1H, *J* = 1.8 Hz), 7.84 – 7.77 (m, 2H), 7.58 (d, 1H, *J* = 2.0 Hz), 7.44 (d, 1H, *J* = 2.0 Hz), 6.86 – 6.79 (m, 2H), 4.71 – 4.60 (m, 4H), 3.88 (s, 3H). **^{13}C NMR (126 MHz, D_2O, 25 °C) δ** 168.0, 152.8, 136.4, 131.7, 123.7, 122.7, 117.5, 114.7, 62.6, 48.5, 48.5, 35.7, 35.7. **ESI-HRMS m/z:** calculated for $C_{13}H_{16}N_3O_2^+$ (M)$^+$: 246.1237, found 246.1235.

6.2 General reductive amination procedure.

In a 50 mL sealable round-bottom glass flask a solution of glycoside **2** or **3** (0.5 mmol) in 5 mL of dimethyl sulfoxide/acetic acid (7:3; v:v) was treated with **2AB** or **GlTag** (0.5 mmol) and stirred at 65 °C for 3 h. Then, $NaBH_3CN$ (88.0 mg 1.4 mmol) was added at 65 °C and the mixture was stirred for 16 h at 65 °C. The solution was cooled down to room temperature and the solvent was removed via lyophilization.

6.3 2AB-GlcNAc (8).

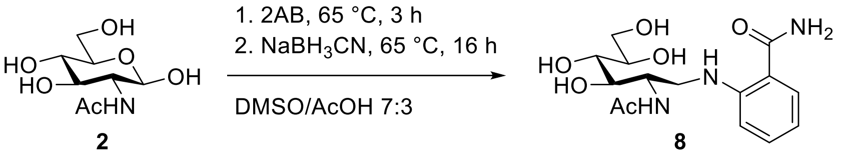

2 **8**

Compound **8** was prepared as described in the general reductive amination procedure starting from GlcNAc **2** (110.6 mg, 0.5 mmol) and **2AB** (67.6 mg, 0.5 mmol). The solid residue was purified via silica gel column chromatography (n-Butanol/Ethanol/H_2O/Acetic acid 5:3:1:0.01 v:v:v:v ratio) furnishing **8** (80.2 mg, 47 %) as a white solid.

^{1}H NMR (500 MHz, D_2O) δ 7.48 (dd, 1H, *J* = 7.9, 1.6 Hz), 7.37 (ddd, 1H, *J* = 8.6, 7.2, 1.6 Hz), 6.89 (dd, 1H, *J* = 8.5, 1.0 Hz), 6.72 (td, 1H, *J* = 7.5, 1.0 Hz), 4.16 (dt, 1H, *J* = 9.8, 5.0 Hz), 3.83 (dd, 1H, *J* = 5.4, 3.1 Hz), 3.70 (dd, 1H, *J* = 11.6, 3.2 Hz), 3.66 (ddd, 1H, *J* = 7.5, 6.3, 3.2 Hz), 3.57 – 3.45 (m, 3H), 3.18 (dd, 1H, *J* = 13.9, 9.3 Hz), 1.87 (s, 3H). **^{13}C NMR (126 MHz, D_2O) δ** 174.2, 174.1, 148.3, 133.5, 129.1, 116.9, 116.2, 113.4, 71.4, 71.1, 69.4, 62.6, 50.8, 43.6, 21.9. **ESI-HRMS m/z:** calculated for $C_{15}H_{23}N_3O_6Na$ (M+Na)$^+$ 364.1479, found 364.1493.

6.4 GlTag-GlcNAc (9).

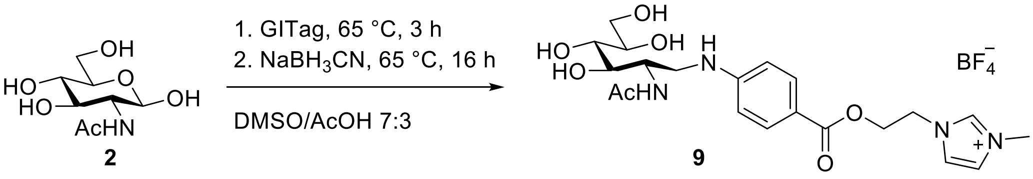

Compound **9** was prepared as described in the general reductive amination procedure starting from GlcNAc **2** (110.6 mg, 0.5 mmol) and **GlTag** (116.5 mg, 0.5 mmol). The solid residue was purified via silica gel column chromatography (methanol/H_2O/acetic acid 7:3:0.04 v:v:v ratio) furnishing **9** (143.4 mg, 57 %) as a white solid.

^{1}H NMR (400 MHz, D_2O) δ 8.84 (s, 1H), 7.81 (dd, 2H, *J* = 8.8, 1.7 Hz), 7.59 (t, 1H, *J* = 1.8 Hz), 7.46 (t, 1H, *J* = 1.8 Hz), 6.77 (dd, 2H, *J* = 8.8, 1.7 Hz), 4.69 – 4.60 (m, 4H), 4.32 – 4.22 (m, 1H), 3.96 (dd, 1H, *J* = 5.6, 3.0 Hz), 3.89 (s, 3H), 3.85 – 3.72 (m, 2H), 3.68 – 3.61 (m, 2H), 3.53 (dd, 1H, *J* = 14.5, 5.1 Hz), 3.35 (dd, 1H, *J* = 14.3, 8.6 Hz), 1.92 (d, 3H, *J* = 1.5 Hz). **^{13}C NMR (126 MHz, D_2O)** δ 174.3, 168.0, 153.3, 136.4, 131.7, 123.7, 122.7, 115.8, 112.1, 71.5, 71.1, 69.1, 62.5, 51.3, 48.5, 43.3, 35.7, 23.2. **ESI-HRMS m/z:** calculated for $C_{21}H_{31}N_4O_7^+$ $(M)^+$ 451.2187, found 451.2191.

6.5 2AB-Lac (10).

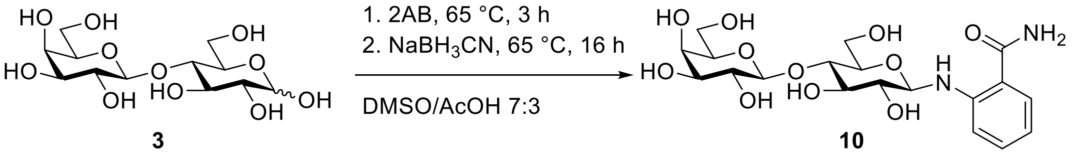

Compound **10** was prepared as described in the general reductive amination procedure starting from Lactose **3** (171.2 mg, 0.5 mmol) and **2AB** (67.6 mg, 0.5 mmol). The solid residue was purified via silica gel column chromatography (n-Butanol/Ethanol/H_2O/Acetic acid 5:3:1:0.01 v:v:v:v ratio) furnishing **10** (133.5 mg, 58 %) as a white solid.

^{1}H NMR (500 MHz, D_2O) δ 7.50 (dd, 1H, *J* = 7.9, 1.6 Hz), 7.38 (ddd, 1H, *J* = 8.6, 7.2, 1.6 Hz), 6.85 (dd, 1H, *J* = 8.5, 1.1 Hz), 6.76 – 6.69 (m, 1H), 4.41 (d, 1H, *J* = 7.7 Hz), 4.05 (ddd, 1H, *J* = 8.2, 5.4, 4.0 Hz), 3.89 – 3.74 (m, 5H), 3.65 (dd, 1H, *J* = 11.9, 5.9 Hz), 3.59 – 3.53 (m, 4H), 3.47 (dd, 1H, *J* = 10.0, 7.7 Hz), 3.39 (dd, 1H, *J* = 12.9, 4.1 Hz), 3.14 (dd, 1H, *J* = 12.9, 8.2 Hz). **^{13}C NMR (126 MHz, D_2O) δ** 174.3, 148.6, 133.6, 129.1, 116.8, 116.1, 113.2, 103.0, 79.2, 74.9, 72.5, 71.1, 71.0, 70.6, 70.1, 68.4, 62.0, 60.7, 45.5. **ESI-HRMS m/z:** calculated for $C_{19}H_{30}N_2O_{11}Na$ $(M+Na)^+$ 485.1742, found 485.1757.

6.6 GlTag-Lac (11).

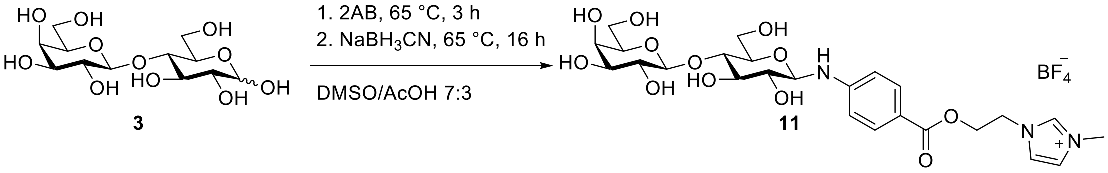

Compound **11** was prepared as described in the general reductive amination procedure starting from Lactose **3** (171,2 mg, 0.5 mmol) and **GlTag** (116.5 mg, 0.5 mmol). The solid residue was purified via silica gel column chromatography (methanol/H_2O/acetic acid 7:3:0.04 v:v:v ratio) furnishing **11** (151.2 mg, 46 %) as a white solid.

^{1}H NMR (500 MHz, D_2O) δ 8.83 (t, 1H, *J* = 1.7 Hz), 7.86 – 7.78 (m, 2H), 7.57 (t, 1H, *J* = 1.7 Hz), 7.44 (d, 1H, *J* = 1.7 Hz), 6.81 –

6.74 (m, 2H), 4.67 – 4.60 (m, 4H), 4.51 (d, 1H, *J* = 7.8 Hz), 4.12 (dt, 1H, *J* = 7.9, 4.6 Hz), 3.97 – 3.90 (m, 3H), 3.89 – 3.83 (m, 5H), 3.73 (dd, 1H, *J* = 11.9, 5.4 Hz), 3.70 – 3.63 (m, 4H), 3.56 (dd, 1H, *J* = 10.0, 7.8 Hz), 3.46 (dd, 1H, *J* = 13.5, 4.4 Hz), 3.28 (dd, 1H, *J* = 13.5, 8.0 Hz). **^{13}C NMR (126 MHz, D_2O) δ** 168.0, 153.5, 136.4, 131.7, 123.7, 122.7, 115.9, 112.2, 103.0, 79.4, 75.0, 72.5, 71.1, 71.0, 70.4, 70.1, 68.4, 62.5, 62.0, 60.7, 48.6, 45.2, 35.7. **ESI-HRMS m/z:** calculated for $C_{25}H_{38}N_3O_{12}^+$ (M)$^+$ 572.2450, found 572.2474.

7. ^{1}H-NMR and ^{13}C-APT NMR

^{1}H NMR (500 MHz, D_2O, 25 °C) of 1.

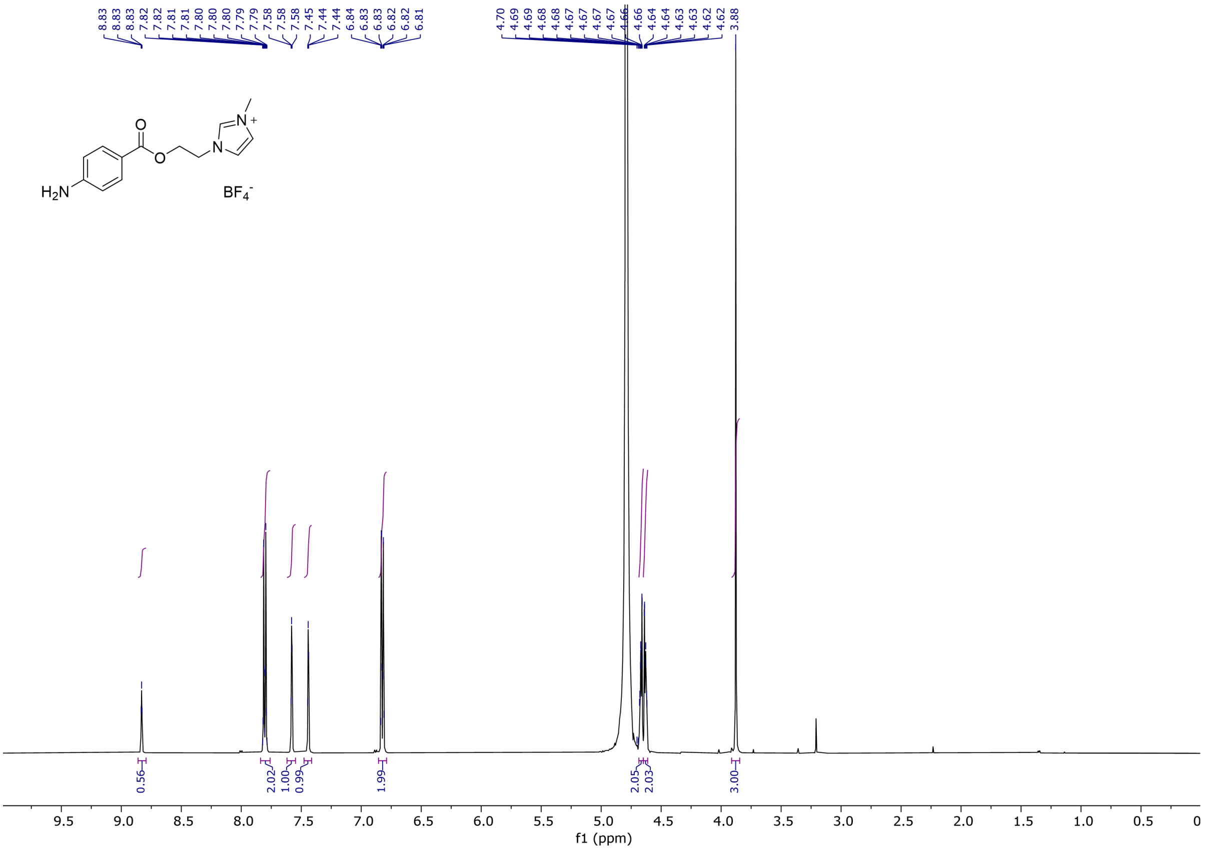

^{13}C APT NMR (126 MHz, D_2O, 25 °C) of 1.

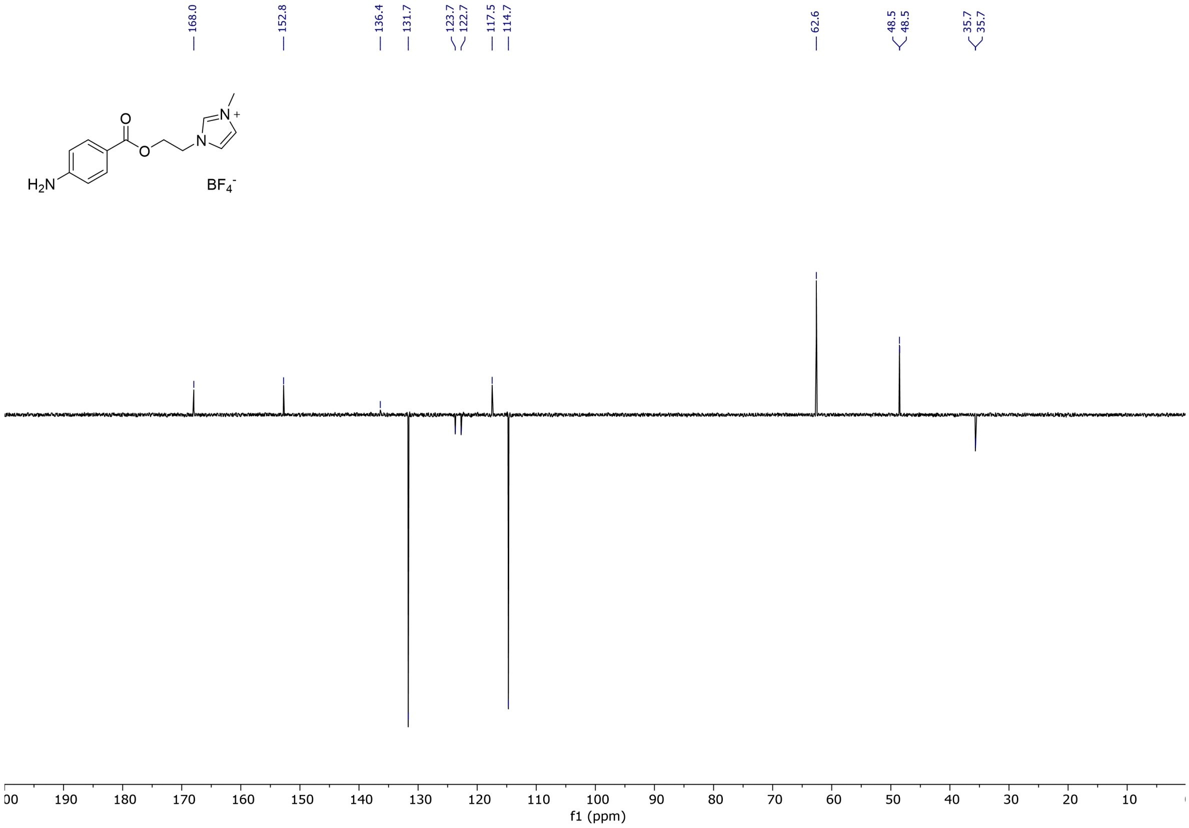

^{1}H NMR (500 MHz, D_2O, 25 °C) of 8.

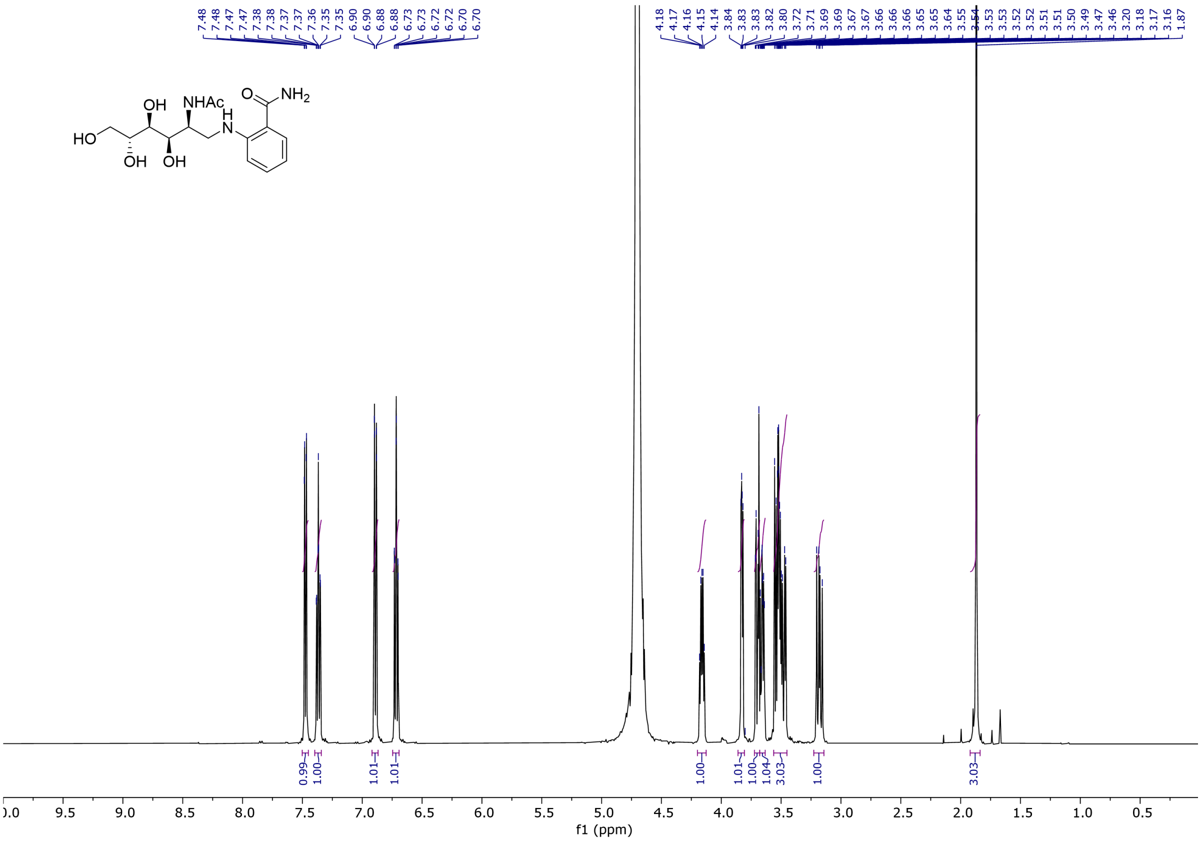

^{13}C APT NMR (126 MHz, D_2O, 25 °C) of 8.

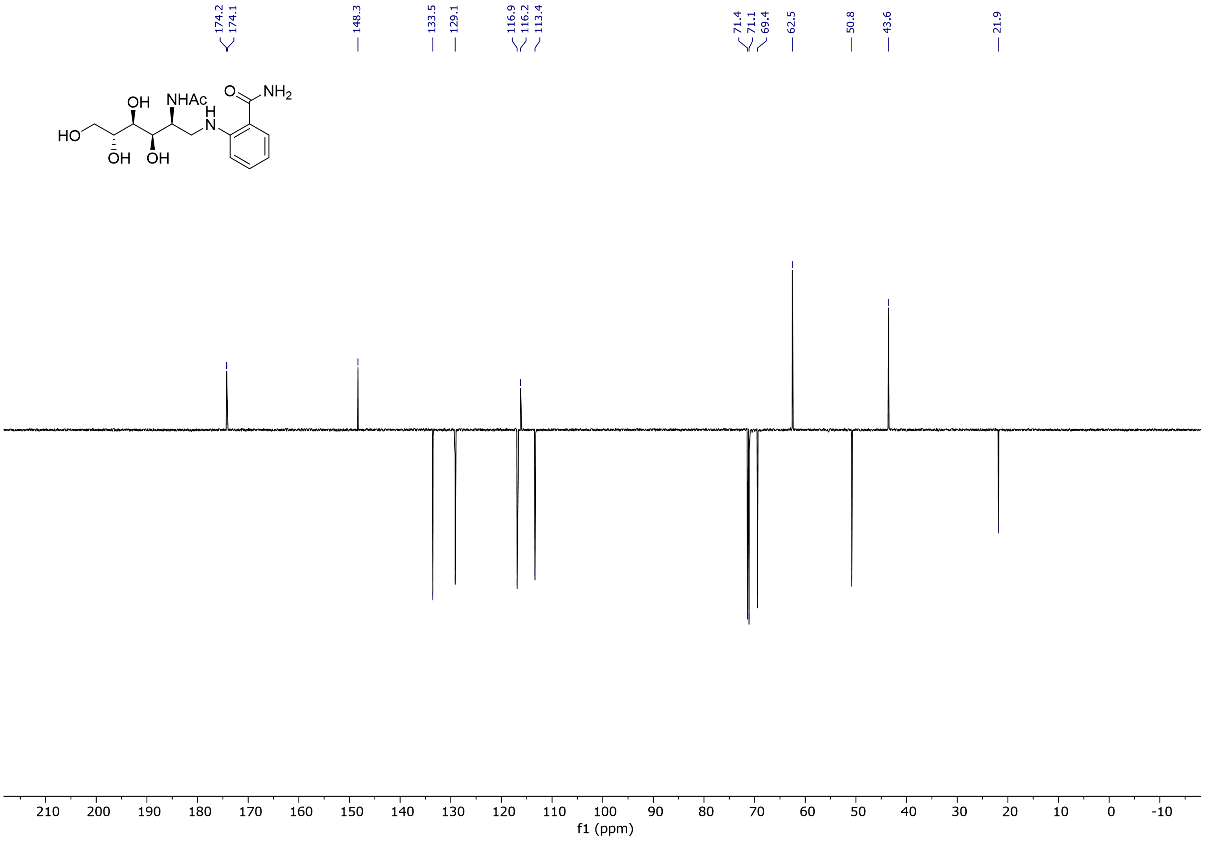

^{1}H NMR (400 MHz, D_2O, 25 °C) of 9.

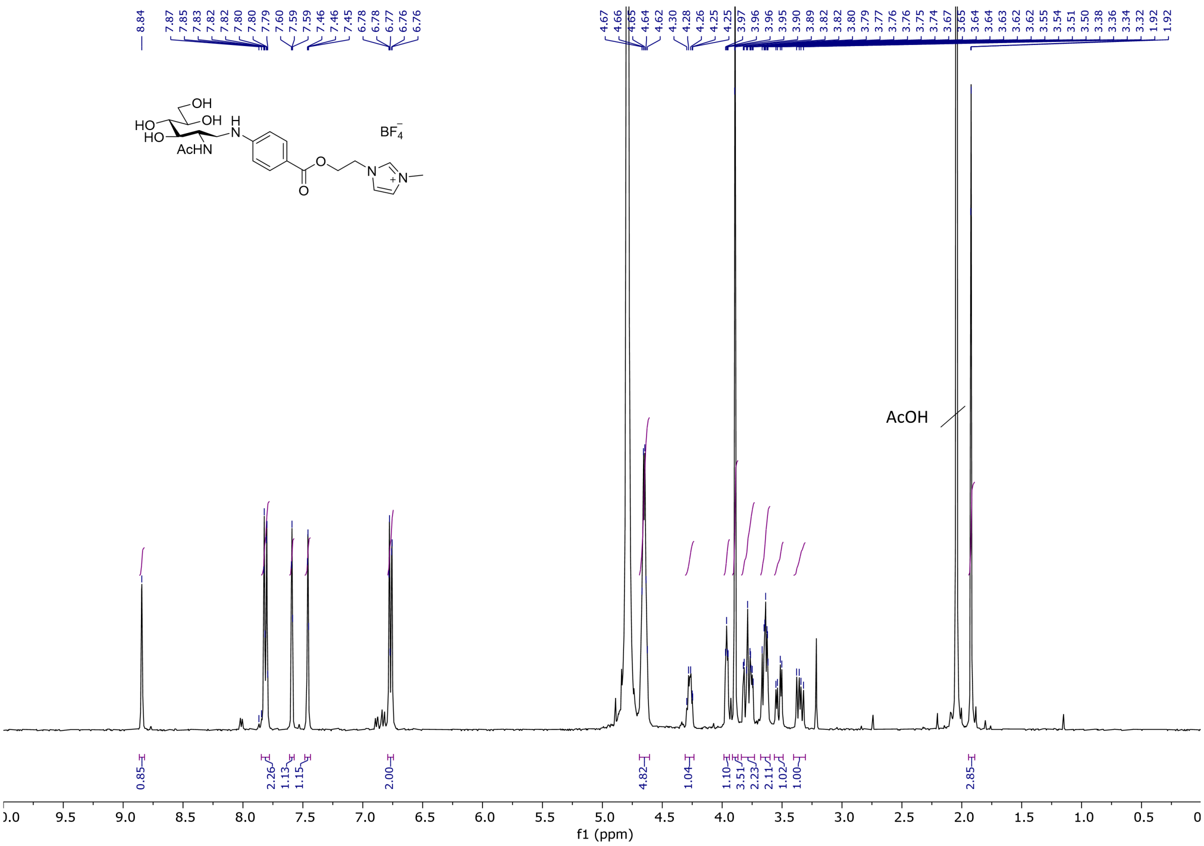

^{13}C APT NMR (126 MHz, D_2O, 25 °C) of 9.

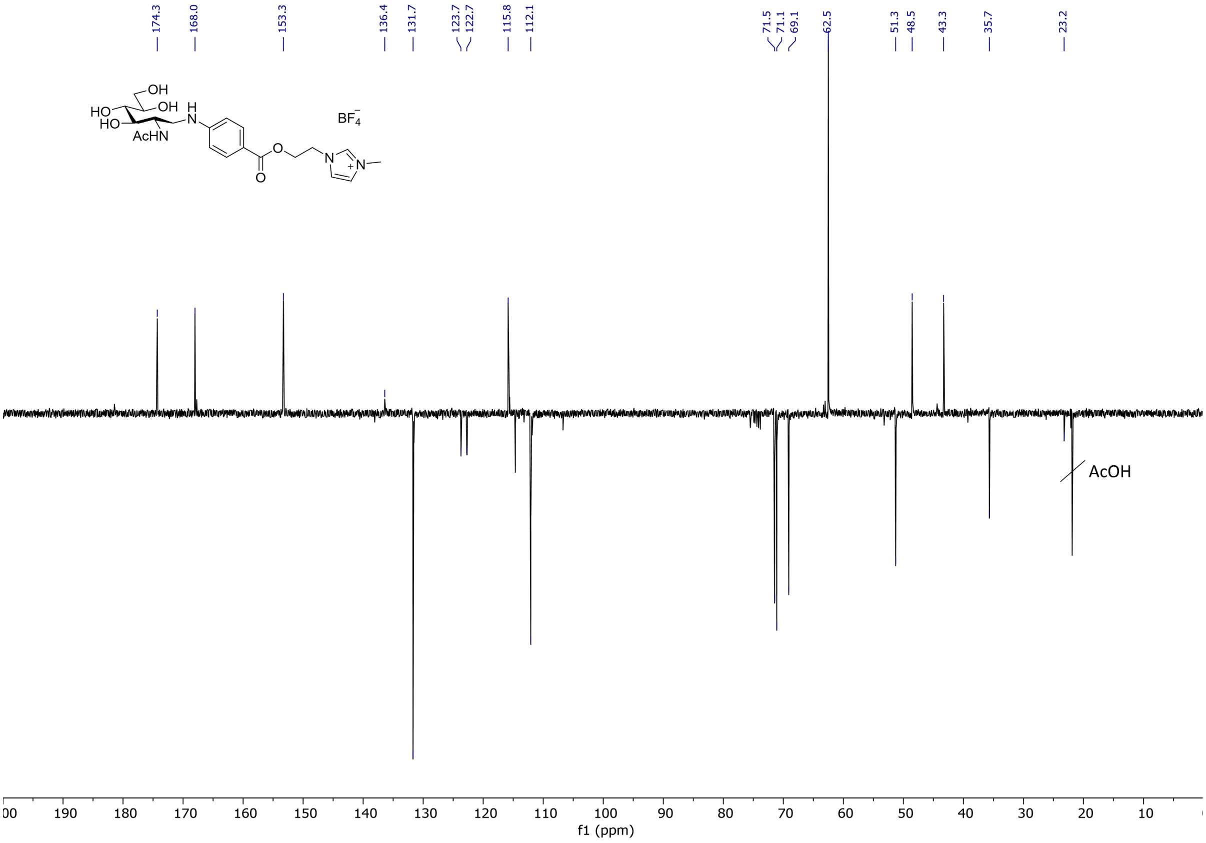

^{1}H NMR (500 MHz, D_2O, 25 °C) of 10.

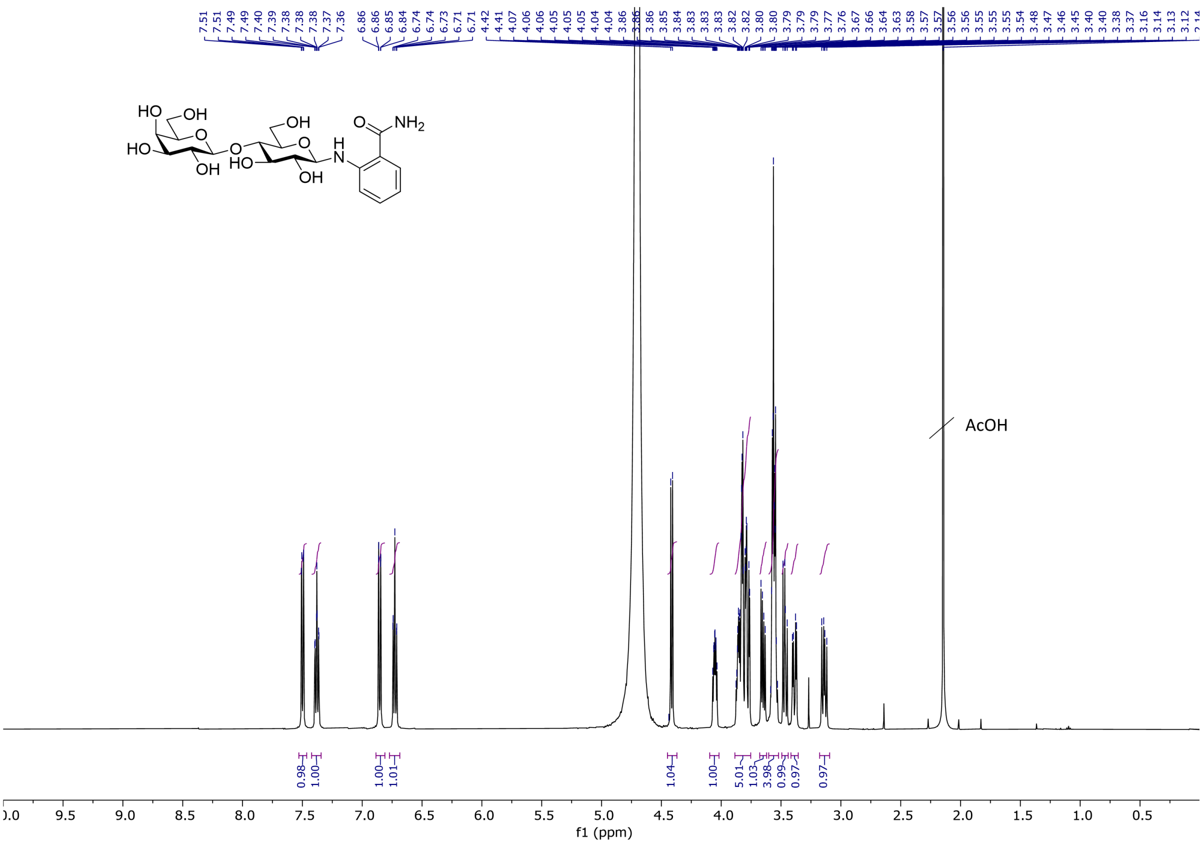

^{13}C APT NMR (126 MHz, D_2O, 25 °C) of 10.

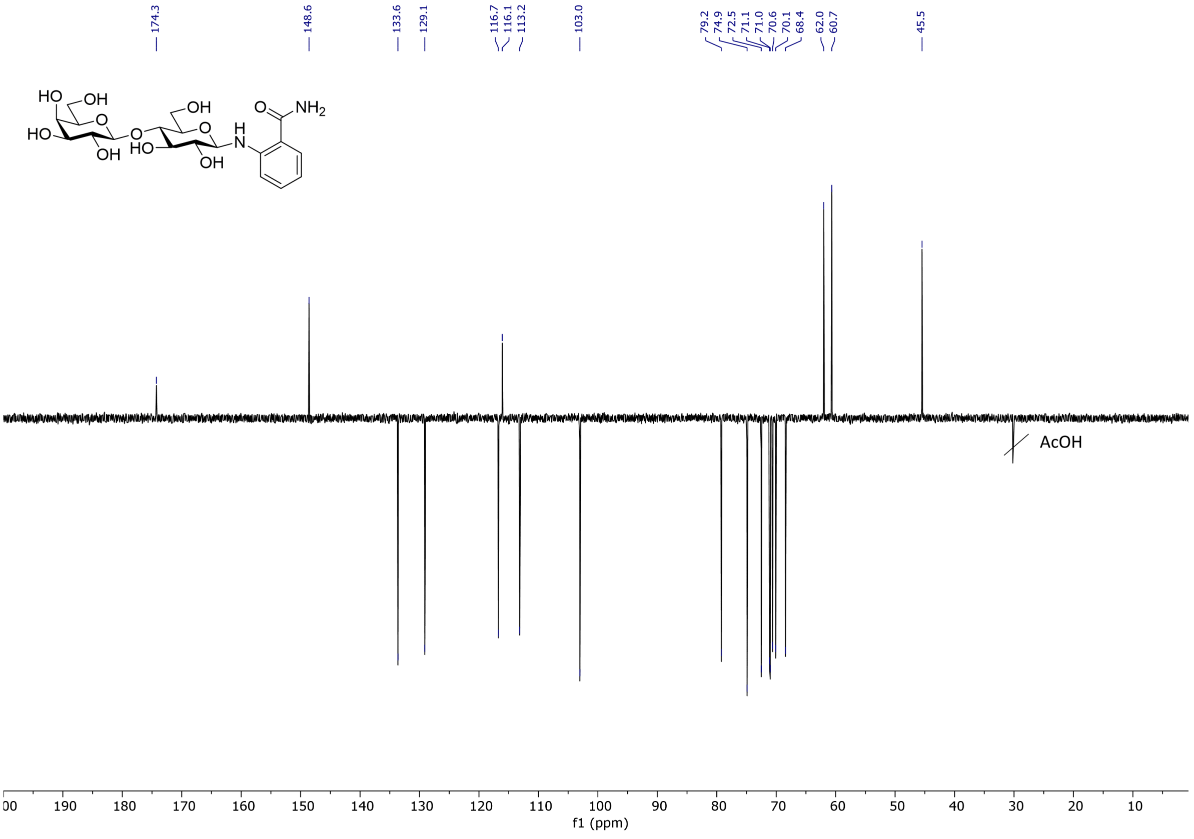

^{1}H NMR (500 MHz, D_2O, 25 °C) of 11.

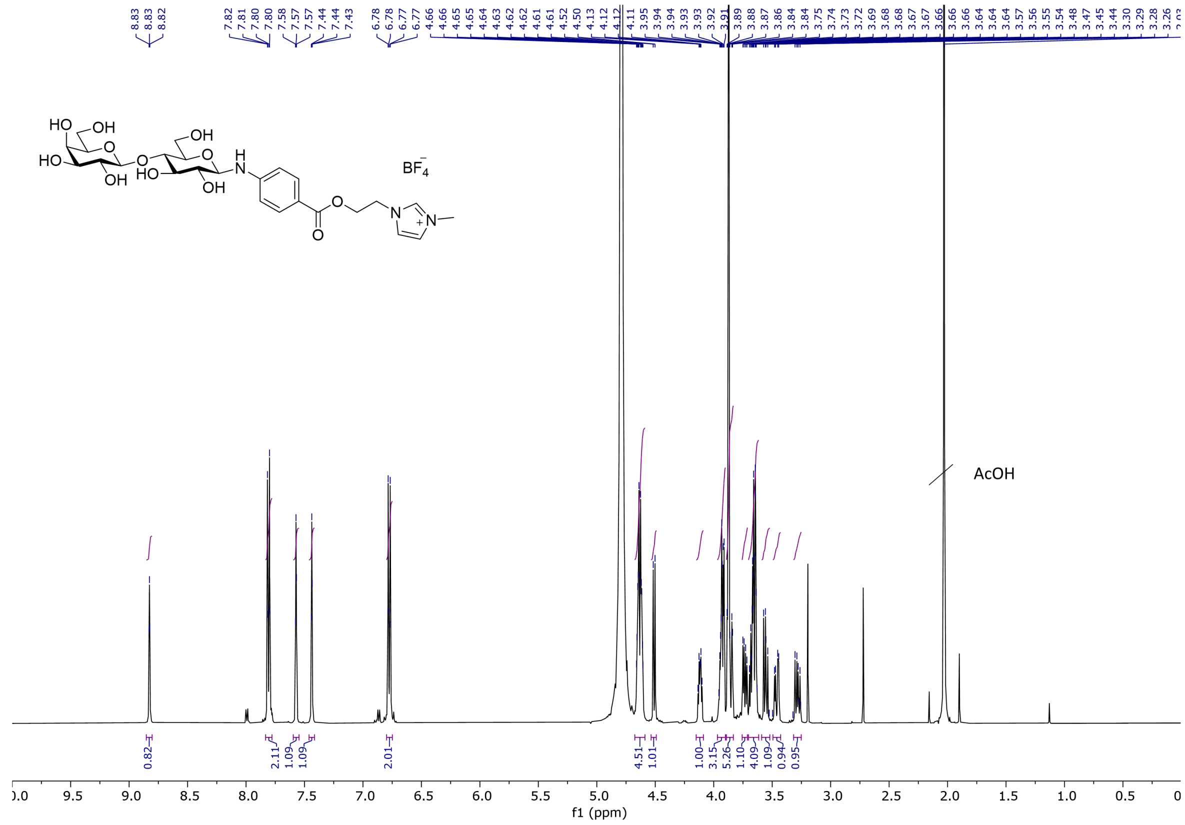

^{13}C APT NMR (126 MHz, D_2O, 25 °C) of 11.

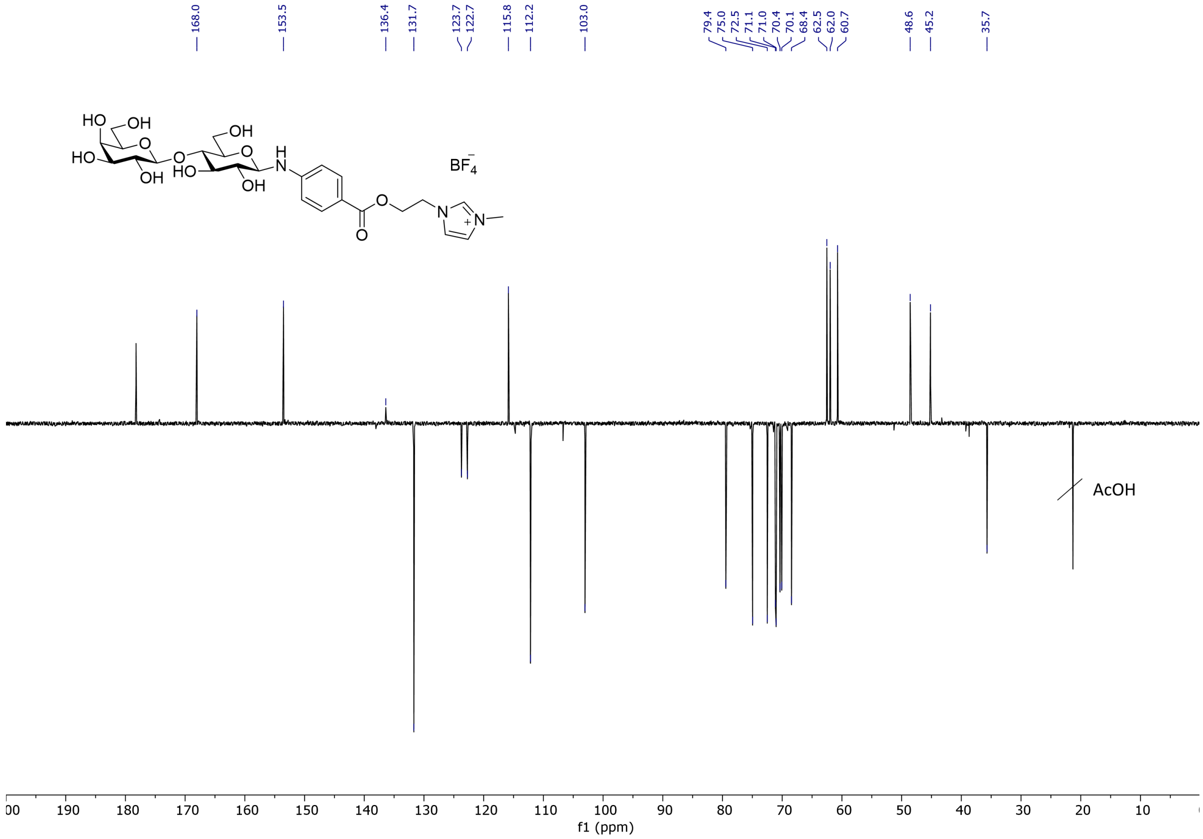

Supplement: CC-057-D1CC02100A-s001 [file CC-057-D1CC02100A-s001.pdf]
